# Fungal Screening on Olive Oil for Extracellular Triacylglycerol Lipases: Selection of a *Trichoderma harzianum* Strain and Genome Wide Search for the Genes

**DOI:** 10.3390/genes9020062

**Published:** 2018-01-25

**Authors:** Miguel Angel Canseco-Pérez, Genny Margarita Castillo-Avila, Bartolomé Chi-Manzanero, Ignacio Islas-Flores, Max M. Apolinar-Hernández, Gerardo Rivera-Muñoz, Marcela Gamboa-Angulo, Felipe Sanchez-Teyer, Yeny Couoh-Uicab, Blondy Canto-Canché

**Affiliations:** 1 Unidad de Biotecnología, Centro de Investigación Científica de Yucatán, A.C., Calle 43 No. 130 X 32 y 34, Col. Chuburná de Hidalgo, C.P. 97205 Merida, Mexico; miguel.canseco@cicy.mx (M.A.C.-P.); gmca1983@gmail.com (G.M.C.-A.); bchim@cicy.mx (B.C.-M.); mizramax@gmail.com (M.M.A.-H.); mmarcela@cicy.mx (M.G.-A.); dirgen@cicy.mx (F.S.-T.); 2 Centro de Estudios Tecnológicos del Mar No. 17, km 1.5 Carr. Antigua Chelem, C.P. 97320 Yucalpetén, Mexico; 3 Unidad de Bioquímica y Biología Molecular de Plantas, Centro de Investigación Científica de Yucatán, A.C., Calle 43 No. 130 X 32 y 34, Col. Chuburná de Hidalgo, C.P. 97205 Merida, Mexico; islasign@cicy.mx; 4 Departamento de Ingeniería Química y Bioquímica, Tecnológico Nacional de México, Campus Instituto Tecnológico de Mérida, Km. 5 Carr. Mérida-Progreso S/N, C.P. 97118 Merida, Mexico; grivera@itmerida.mx; 5 División de Ingeniería Bioquímica, Instituto Tecnológico Superior de Purísima del Rincón. Blvd del Valle #2301, Guardarrayas, C.P. 36413 Purísima del Rincón, Guanajuato, Mexico; yeny.couoh@tecpurisima.edu.mx

**Keywords:** enzymes, lipases, triacylglycerol lipases, true lipases, olive oil induced lipases, protein bioinformatic analyses

## Abstract

A lipolytic screening with fungal strains isolated from lignocellulosic waste collected in banana plantation dumps was carried out. A *Trichoderma harzianum* strain (B13-1) showed good extracellular lipolytic activity (205 UmL^−1^). Subsequently, functional screening of the lipolytic activity on Rhodamine B enriched with olive oil as the only carbon source was performed. The successful growth of the strain allows us to suggest that a true lipase is responsible for the lipolytic activity in the B13-1 strain. In order to identify the gene(s) encoding the protein responsible for the lipolytic activity, in silico identification and characterization of triacylglycerol lipases from *T. harzianum* is reported for the first time. A survey in the genome of this fungus retrieved 50 lipases; however, bioinformatic analyses and putative functional descriptions in different databases allowed us to choose seven lipases as candidates. Suitability of the bioinformatic screening to select the candidates was confirmed by reverse transcription polymerase chain reaction (RT-PCR). The gene codifying 526309 was expressed when the fungus grew in a medium with olive oil as carbon source. This protein shares homology with commercial lipases, making it a candidate for further applications. The success in identifying a lipase gene inducible with olive oil and the suitability of the functional screening and bioinformatic survey carried out herein, support the premise that the strategy can be used in other microorganisms with sequenced genomes to search for true lipases, or other enzymes belonging to large protein families.

## 1. Introduction

Lipases are serine hydrolases defined as triacylglycerol acyl hydrolases (EC 3.1.1.3), which hydrolyze glycerol esters of long chain fatty acids (water insoluble). Many enzymes have activity on water soluble medium-chain triacylglycerols (<12 carbon atoms), long chain monoacyl glycerols or long-chain nitrophenyl acyl esters but are not able to act against triacylglycerols (TAGs) with long-chain fatty acids, such as vegetable oils. Those lipases are not considered true lipases but they are esterases (EC 3.1.1.1), which hydrolyze esters of short chain fatty acids (water soluble). For lipase catalysis there is a phenomenon called interfacial activation, which does not occur in esterases [1]. Substrate emulsion generates a lipid interface that activates the lipase: a loop which covers the active site (lid) changes conformation and the enzyme shifts to the open conformation, where the active site is accessible to the substrates [2]. In addition to hydrolyses, lipases are involved in conversion reactions in non-aqueous media: esterification, interesterification, transesterification, alcoholysis, acidolysis and aminolysis [1,3].

Lipases have been known since 1930 and have captured the interest of scientists and industrial businessmen to the present day. These enzymes are used in the detergent industry, in the organic synthesis of pharmaceuticals, pesticides and insecticides, the production of emollient for personal care in cosmetics and more recently in transesterification reactions for biodiesel production and in the emerging oil and fat industry, for example production of cocoa butter equivalent (chocolate) and human milk fat substitute [1,4,5]. Recently, Daiha et al. [4] reviewed contemporary research publications and patent applications dealing with lipases and concluded that these enzymes are of much relevance in modern industry; however, further research is still needed to reach their full potential. 

Lipases are ubiquitous in living beings. In particular, microbial lipases are of great importance and have diverse commercial applications [6]. Bacteria, yeasts, filamentous fungi and a few protozoa produce extracellular lipases for the digestion of lipid materials [1]. Screening for extracellular lipases is currently performed on isolated microorganisms, by microbial genome-wide mining, or by metagenomic mining. Lipolytic microorganisms have been isolated or identified in collections from soils, plant matter, plant compost, oleaginous fruits, domestic vegetable oil wastes, pig manure, organic dumps, garbage collectors and industrial effluents, among many other sources. Microbes are highly successful in adapting and surviving in a wide range of environments, by exploiting their trophic niche; thus, the ability to secrete enzymes is of great survival value. 

There are many screening methods for lipolytic activity [7]. The most common is solid media added with different lipid substrates and lipase activity can be detected as clear or turbid zones around the colonies or by the production of crystals on the agar surface. Combination with Rhodamine B has proved to be a fast method; Rhodamine B binds fatty acids, mono- and diglycerides and develops fluorescence under ultra violet (UV) light [1,7]. Tributyrin-agar plate is a widely used method, but this substrate cannot distinguish between true lipases and esterases. Olive oil-agar plates are a good test for screening true lipase-positive colonies [1,8]. Lipase production is often induced by the presence of vegetable oils and the best results have been obtained with olive oil [8,9,10,11].

In the present work, fungi isolated from banana plantation waste were screened for production of extracellular lipases on olive oil-Rhodamine B plates. *Trichoderma harzianum* and *Trichoderma longibrachiatum* were identified as the most prominent producers, the former being the best strain in our collection. We hypothesize that the lipase responsible for the extracellular activity on olive oil is a true lipase. Ülker et al. [12] performed the first characterization of the extracellular lipolytic activity of *T. harzianum*. They found that the activity is optimum at 40 °C and that it is thermostable, showing 55% of activity when it was incubated at 50 °C and 20% with incubation at 70 °C. Coradi et al. [9] precipitated the protein and found that the lipolytic enzyme worked on long-chain TAGs as triolein and olive oil, i.e., it is a true lipase. 

To date there is no description regarding genes for lipases in *T. harzianum*. The goal of this work is to identify the gene or genes that encode the protein responsible for the extracellular lipolytic activity of this fungus on olive oil.

## 2. Materials and Methods

### 2.1. Isolation of Lipolytic Microorganisms

Fungi were isolated from lignocellulolytic residues collected on (a) a garbage dump and (b) plant debris from a banana plantation in Tabasco, Mexico. Decaying vegetal materials were sampled and transported to the laboratory in paper bags and plastic boxes covered inside with sterile wet paper (humid chamber). Fungi were isolated under a stereoscope (Motic, Richmond, BC, Canada) using an inoculating teasing needle and plate (Sigma Aldrich, Saint Louis MO, USA) on Malt extract agar medium (Sigma Aldrich) with 250 µg L^−1^ amikacin (Sigma Aldrich) subcultures [13]. Only fungal cultures showing homogenous growth (i.e., pure strains) were considered for further analysis. 

### 2.2. Screening of Lipolytic Fungi 

Screening was performed on selective Potate Dextrose Agar (PDA) (DIBICO, Cuautitlan Izcalli, Mexico) supplemented with 1% (*v*/*v*) olive oil (Selecto Choice, Spain) and 0.001% (*w*/*v*) of Rhodamine B (Sigma Aldrich) [14]. The medium was emulsified by mixing with a polytron homogenizer Ultra-turrax T25 (IKEA, Staufen, Germany) and poured into Petri dishes. A disk (1 cm diameter) of each fungus grown on PDA medium (72 h) was inoculated on the center of the Petri dish containing the solid medium. Each fungus was independently inoculated in triplicate and grown at 28 °C. The presence of free fatty acids was detected by UV Light [1,7]. The level of lipase production was evaluated by monitoring on UV transilluminator (360 nm) (UVP Analytic Jena Company, Upland, CA, USA) every 12 h over a period of 48 h.

Four categories were defined, based on the fluorescence intensity and the area of the fluorescent colony. Category 0 was assigned with “−” for all strains without fluorescent halo, category 1 (+) includes strains with poor fluorescence and growth, category 2 (++) includes good growth and moderate fluorescence and category 3 (+++) includes strains with high fluorescence and growth [8,15].

### 2.3. Lipase Production by Submerged Fermentation

Fungal strains which showed stronger fluorescence on Rhodamine B medium were grown in liquid medium as described in Maia et al. [16] with 1% (*v*/*v*) olive oil as sole carbon source. Fungal strains were grown on PDA for 72 h and two cylinders of 0.5 cm diameter each were macerated with 1 mL sterile water and inoculated in 125 mL of culture medium. Fungal cultures were incubated with constant shaking at 180 rpm at 30 °C in incubator shaker 3532 (LAB-LINE Instruments, Melrose Park, IL, USA) Samples were harvested every 24 h over a period of 8 days.

Cell-free extracellular proteins were obtained by centrifuging the sample at 15,000 rpm for 15 min at 4 °C in centrifuge 5810R (Eppendorf, Hamburg, Germany). Supernatants were recovered to measure lipase activity.

Lipolytic activity was determined spectrophotometrically on p-nitrophenyl palmitate (pNPP; Sigma-Aldrich) as substrate. Buffer added with pNPP (1.2 mL) was prewarmed to 37 °C. Reaction was started by adding 50 µL of enzyme extract and incubation for 15 min at 37 °C. The free p-nitrophenol (pNP) was monitored at 410 nm in Genesys 10s UV-VIS (Thermo Spectronic, Madison, WI, USA) [17]. A blank sample was always used containing medium described in Maia et al. [16] instead of enzyme solution. The molar extinction coefficient of ε = 15,000 M^−1^ cm^−1^ for pNP at 410 nm [17,18] was used for calculus. One enzymatic unit was defined as the amount of the enzyme that releases one mmol of pNP in one minute under the assay conditions. 

### 2.4. Molecular Identification of Selected Lipolytic Fungi Isolated Here

To obtain DNA, 500 mg of mycelia were extracted following the procedure of Johanson et al. [19]. Internal transcribed spacer (ITS) regions were amplified using as template 10 ng of genomic DNA and the ITS1 and ITS3 primers [20]. Amplicons were gel-purified and sent to LANBAMA (IPICyT, San Luis Potosi, Mexico) for both strands sequencing. Bioedit software was used for sequence edition [21], removing the sequence ends to eliminate noise (approx. 20 bp in each end). Antisense sequences were submitted to UGENE software to convert them into their respective reverse complement sequences [22]. To confirm ITS sequence, for each fungus the sense and the reverse complement were aligned with MUSCLE [23] and manually checked. For each case the consensus sequence was used as query to search by BLASTn [24] in the fungal non-redundant NCBI database [25] and TrichOkey2 [26] specific database for identification of *Hypocrea* and *Trichoderma* species. In each case, the first hit in the list was retrieved and data were manually analyzed.

### 2.5. Searching of Genes of True Lipases in Trichoderma harzianum Genome 

The deduced proteome of *T. harzianum* in the JGI genome portal [27] was searched for lipases, followed by refining the quest for true lipases with the keywords triglyceride lipase, triacylglycerol lipase, and lipase class 3. 

To support the quest of true lipases, BLASTp in the *T. harzianum* deduced filter models of proteins was performed by using as queries the amino acid sequences of reported lipases with activity on long-chain triacylglycerydes (vegetable oils): *Fusarium graminearun* FGL1 AAQ23181 [28], *Fusarium heterosporum* AAB34680 [29]; and the long-chain triacylglyceryde lipases enlisted in the US 9476008 B2 patent [30]: *Rhizomucor miehei* (P19515), *Thermomyces lanigunosus* (CAB58509), *Candida antarctica* LipA (2VEO) and LipB (P41365) and *Rhizopus oryzae* (AER14043).

For each protein hit retrieved, the model name, the scaffold location of their genes in the *T. harzianum* portal and their automatic annotations at portal were recorded. 

### 2.6. In Silico Analysis of Trichoderma harzianum Putative True Lipases

Lipases found in *T. harzianum* with the keywords triglyceride lipase, and those annotated as secreted lipases were selected for further bioinformatic analyses. Amino acid sequence in Fasta format was downloaded for each of the retrieved hits. Lipase candidates were examined at different platforms to search domains/motif information: the Common Domains database in the non-redundant fungal database at NCBI [31], the Pfam databases using the sequence search tool [32], the HHpred (Homology detection and structure prediction by HMM-HMM) [33] and the InterPro database [34].

For identification of extracellular lipases, amino acid sequences were analyzed with SignalP 4.1 server [35] to predict N-terminal secretion signal. Prediction of transmembrane helices was performed with TMHMM 2.0 server [36]. The putative location in the cell was predicted with WoLF PSORT [37]. Molecular size was in silico calculated with Protein Molecular Weight Calculator—Science Gateway [38] and ProtParam tool [39]. 

BLASTp 2.6.0 was performed for rapid assignment [31,40]. Gene Ontology categories, i.e., Biological Process, Molecular Function and Cellular Component were retrieved from the different bioinformatics tools used to search homology, conserved domains, motifs and signatures (Pfam, InterPro, Superfamily, Protein Database, etc.). Each selected candidate was also examined in the genomic portal (clicking on the ID number) to search automatic annotations.

The complete amino acid sequence of each candidate was submitted by BLASTp to the LED (Lipase Engineering Database) [41] and ESTHER Database (ESTerases and alpha/beta-Hydrolase Enzymes and Relatives) [42] to inference class, parent family and probable function through the use of annotation transfer. 

### 2.7. Phylogenetic Analysis 

A list of representative fungal lipases was taken from Yadav et al. [6] and the amino acid sequence from each was downloaded from the GenBank. The putative true lipases retrieved in this study from the deduced proteome of *T. harzianum* and their characterized homologues or best hits in PSI-BLAST [43]: *Tolypocladium ophioglossoides* (KND87948), *Metarhizium anisopliae* (KFG82987), *Nectria haematococca* (CAC19602), *Purpureocillium lilacinum* (XP_018176823), *N. haematococca* (XP_003053404), *Claviceps purpurea* (CCE34466), *Fusarium graminearum* (XP_01138453), *Hirsutella minnesotensis* (KJZ78693), *Fusarium oxysporum* (EWZ47945), *Saccharomyces cerevisiae* (AAA50367), *T. ophioglossoides* (KND93486), *Talaromyces cellulolyticus* (GAM42581), *Colletotrichum fioriniae* (XP_007591165), *T. ophioglossoides* (KND89897), *S. cerevisiae* (AJS39941), *P. lilacinum* (OAQ83707) and *Drechmeria coniospora* (ODA76916). The known true lipases (from literature) mentioned above were included, as well as Lip1 (P20261) and Lip5 (P32949) from *Candida rugosa* [44], the proteins Lip1 (EAA67628, ACE80261 and XP_001800960) from the pathogenic fungi *Gibberella zea* [45], *Blumeria graminis* [46] and *Stagonospora nodorum* [47], *Malassezia restricta* MrLip1 (ALG03641) [48], *Malassezia globosa* (XP_001732206) [49], *Acremonium alcalophilum* LipA (P0CT91) [50], the *S. cerevisiae*’s Tgl3p (AJS99689) and Tgl5p (DAA10860) which are the major TAG lipases in this yeast [51], the *Ophiostoma piceae* sterol esterase (4UPD) [52] and the esterase *Bjerkandera adusta* (APW29213), the latter with no activity on long-chain glycerides [53] which was included as one reference for non-true lipases. The lipases CAA00250 from *R. miehei* and Lip1 and Lip5 from *C. rugosa* are fungal phylogenetic references [44].

A multi-alignment was performed in MAFFT [54] with default parameters. A phylogenetic tree was constructed using the neighbor-joining method [55] with 500 bootstrap.

### 2.8. In Silico Modeling

Three-dimensional modeling was performed with SWISS-MODEL [56], HHpred [57] and I-TASSER [58], using the complete amino sequence of each candidate. The retrieved hit with the highest score was selected in each case as the best model. The catalytic site and the lid domain were identified at IPBA web server [59] by 3D superposition with the best template. Amino acid composition in the conserved motifs in the template were identified and labeled with PyMOL Molecular Graphics System program [60] and then identified in the candidate by structure-based and sequence-based comparisons. 

The PSI-BLAST (Position-Specific Iterated) at the NCBI [43] was conducted to search homologues in the protein structure database. 

For TAG lipase which was de novo expressed on olive oil, a structure-based multi-alignment was conducted by ClustalW [61]. The alignment was represented using ESPript [62].

### 2.9. RT-PCR Amplification of Lipases 

To validate the selection of putative true lipases and to identify the gene/protein responsible for the extracellular lipolytic activity of *T. harzianum* strain B13-1 on olive oil, reverse transcription polymerase chain reaction (RT-PCR) was performed on RNA obtained from the fungus grown in olive oil-free and olive oil-containing medium (1% *v*/*v*). 

The nucleotide coding regions of the selected lipases were downloaded from the *T. harzianum* database [27] and sequence primers were designed with the Primer Quest Tool software [63]. The list and expected sizes of the fragments is shown in Table S1. 

Mycelium was harvested at 1, 3, 5 and 7 days. RNA was extracted with TRIzo Reagent (Invitrogen, Carlsbad, CA, USA), pooled and cDNA was synthesized with Maxima First Strand cDNA Synthesis Kit (Thermo Scientific, Vilnius, Lithuania) following supplier’s recommendations. Each reaction mixture (total volume of 15 µL) contained 1× PCR buffer, 2.0 mM MgCl2, 1.6 µM dNTPs, 400 µM of each primer and 1.0 U of GoTaq DNA polymerase (Promega, Madison, Wi, USA); 1 µL of cDNA was used as template per reaction. The PCR parameters were 3 min of 95 °C; followed by 30 cycles of 94 °C for 30 s, 53 °C for 30 s and 72 °C for 30 s; and a final elongation at 72 °C for 5 min. The PCR products were purified from gel using QIAquick Gel Extraction Kit (Qiagen, Germantown, MD, USA); for sequencing the samples were sent to LANBAMA. Sequences were edited as above and then used for BLAST in the *T. harzianum* genome and NCBI non-redundant protein database.

## 3. Results and Discussion

### 3.1. Screening of Lipolytic Fungi Using Olive Oil

A total of 18 fungal strains isolated from banana lignocellulosic wastes were tested on lipase secretion assay (Table 1). 

All fungal strains were able to grow on solid medium supplemented with olive oil. Thirteen of them were positive on Rhodamine B plates (Table 1). The most active (category 3) were B13-1, B13-3 strains which were considered for further studies (Figure S1). The fluorescence was associated with the mycelia and no clear halos were observed. Many reports on fungal lipolytic strains found no halos around colonies but the mycelia were fluorescent [8,64,65,66,67]. The phenotype found here therefore, is common in filamentous lipolytic fungi tested on olive oil-Rhodamine B medium. Observation of hydrolysis halos is more common in bacteria [68,69]. 

ITS-sequence-based BLASTn in the non-redundant fungal database at NCBI, shows that fungal strain B13-1 has 94% similarity with *T. harzianum* (98% coverage, Evalue 0.0) while B13-3 strain has 98% similarity with *T. longibrachiatum* (98% coverage, Evalue 0.0). *Trichoderma* species have been previously identified in screening of lipolytic fungi from effluents collected in dumpsites with palm oil mill residue [3], slaughterhouses and dairy industries [68], as well as soils contaminated with waste vegetable oils [8]. In fact, Nwuche and Ogbonna [3] reported that the highest lipase producing strains in their screening belong to the *Trichoderma* genus. 

### 3.2. Lipolytic Activity Assay

**Figure 1:**
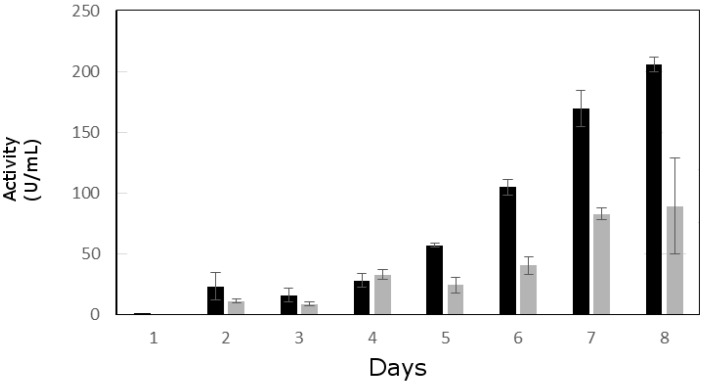
Extracellular lipolytic activity measured with *p*-nitrophenol palmitate. Black bars show the activity in the strain B13-1; gray bars show the activity in the strain B13-3. The data show the standard deviation of three independent samples.

In *T. harzianum* the extracellular lipase activity increased gradually reaching a maximum on the 8th day (205 UmL^−1^). *T. longibrachiatum* showed a similar pattern of extracellular lipolytic activity with gradual increment but maximum activity at the 8th day (109 UmL^−1^) was half that produced by *T. harzianum* B13-1 strain (Figure 1). Lipase activities in both fungi are in the range reported for good lipase producing strains, such as *Penicillium chrysogenum* (205 UmL^−1^) [70] and *R. oryzae* from oil palm fruit (120 UmL^−1^) [71]. To date, only one report with a stronger production of lipolytic activity from *Rhizopus* sp. has been found. This fungus was isolated from contaminated oil and reported 870 UmL^−1^ of lipolytic activity [72].

Since the activity was higher in *T. harzianum* B13-1 than in *T. longibrachiatum* B13-3, we decided to continue with the first strain. Based on the methodology followed in the present work, i.e., screening of lipolytic fungi on olive oil-containing medium and the subsequent production of lipolytic activity by using olive oil as carbon source, together with available information on *T. harzianum* lipase, it is reasonable to propose that the lipolytic activity found in *T. harzianum* B13-1 corresponds to a true lipase.

### 3.3. Searching Genes of True Lipases in Trichoderma harzianum

A survey of the genome was conducted to explore the possibility of identifying the respective gene among the *T. harzianum* TAG lipases. To date, there have been no reports on the lipase genes in this microorganism. The quest in the *T. harzianum* deduced proteome at genomic portal [27] using “lipase” as keyword resulted in 50 hits (Table S2), showing that the lipase family is large in this fungus. The family comprises alpha-beta hydrolases, phospholipases, carbohydrate esterases, isoamyl acetate-hydrolyzing esterase, among others. Four lipases (551811, 87496, 526309 and 514427) are annotated as secreted in the *T. harzianum* genome portal and are therefore plausible candidates to explain the extracellular lipolytic activity measured here in strain B13-1. To expand the list of candidates, the search in the genome for true lipases was performed by using the keywords “triglyceride lipase” and it retrieved 9 hits (IDs 510832, 79895, 92423, 135964, 492160, 77338, 78181, 502433 and 514252). The last four belong to lipase class 3, which is a lipase family of true lipases [25,28]. Lipases with activity on long-chain TAG were used as queries to search for more true lipases in *T. harzianum*: *F. graminearun* FGL1 AAQ23181, *F. heterosporum* AAB34680, *R. miehei* P19515 and *R. oryzae* AER14043 retrieved all of the *T. harzianum* proteins 78181 and 77338; *T. lanigunosus* CAB58509 retrieved these two lipases and the protein 502433. These three lipases are in the list found previously. 

Therefore, the list comprises 13 candidates in the search for the putative true lipase responsible for the extracellular lipolytic activity on olive oil in *T. harzianum*. 

### 3.4. In Silico Characterization of Trichoderma harzianum True Lipases 

BLASTp 2.6.0 was performed for rapid assignment of lipase candidates but for most of them the closest homologue was uncharacterized predicted lipase, or hypothetical protein, arising from genomic sequencing projects. Therefore, this analysis was not of much help in supporting or eliminating candidates. Genomic data is so abundant in the GenBank that usually 50–100 hits corresponded to uncharacterized predicted lipase, although some of the lipase candidates under analysis were retrieved by BLASTp from *T. harzianum* portal using a characterized TAG lipase as query. However, in the huge sequencing data, those queries ranked so far in comparison with many genomic sequences. 

PSI-BLAST increases the opportunities to predict function, especially when *Trichoderma* species are excluded, because the searching result rules out the large list of predicted, hypothetical proteins that come from genomic sequencing data from this genus. Table 2 shows a summary of sequence-based function transfer based on PSI-BLAST.

Although BLASTp hits were not suitable to predict function by homology, the conserved domain searching tool hosted in BLAST server enabled us to find motifs and conserved domains in the protein candidates (Table 2). The analysis in the conserved domain database (CDD) confirms the lipase 3 domain in 77338, 78181, 514252 and 502433, consistent with the automatic annotation for these proteins in the genomic portal of *T. harzianum*. The Lipase 3 domain contains the flip/lid domain (TYITNTIIDLS in 77338, SNLRNFITDVV in 78181, TSTNDKVNDNL in 514252 and TLFEDVLADLT in 502433), the catalytic triad (Ser-His-Asp) and the nucleophilic elbow (GXSXG consensus motif) which is GHSLG in these four lipases. In addition to these domains, 502433 has one AF-4 domain. This domain is present in AF4 and FMR2 nuclear proteins. In *Drosophila* AF4 protein homologue acts in cytoskeleton regulation, segmentation and morphogenesis. 

Since the survey performed in this work is for identifying the protein responsible for extracellular activity in B13-1 strain on olive oil, molecular size and cellular localization were analyzed. One of the putative lipase class 3 gave a negative result for signal peptide in SignalP program (502433) and its size is larger (115 kDa) than expected for extracellular lipases, which ranks from molecular weights less than 20 to 65 kDa [73,74]. The other three class 3 lipases were predicted with signal peptide and as being extracellular, making them good candidates (Table 3).

The analysis in the CDD identified the alpha/beta hydrolase fold on the putative TAG lipases 510832, 92423 and 135964. Top hits at BLASTp retrieved sterol esterases for 510832 and GPI inositol-deacylase PGAP1-like protein for 135964, the last one probably involved in vesicular traffic from endoplasmatic reticulum. 

92423 shares homology with lysosomal acid and other acid lipases. Consistent with intracellular roles, any of these three lipases are predicted extracellular (Table 3). Regarding the other two putative TAG lipases, 79895 and 492160, neither lipase nor hydrolase domains were identified but WD40 and Sec domains in the former and Diaphanous FH2/FH3 domains in the later (Table 2), suggesting roles with other functions than lipases. Deduced sizes are 134 and 198 kDa respectively; it is not prognosticated secretion for these proteins and in agreement, signal peptide was not identified.

The four automatically annotated secreted lipases (514427, 526309, 551811 and 87496), show Abhydrolase fcl21494 domain, containing the catalytic triad Ser-His-Asp and contain the Pfam domain 03583 which is related with a lipase from *C. albicans*. Curiously, one of those proteins, 551811, gave negative prediction of signal peptide with SignalP and WoLF PSORT predicts cytoplasmic localization. A Rossmann-fold NAD(P)H/NAD(P)(+) binding (NADB) domain was identified in 551811, a domain found in numerous redox enzymes. 514427 shares homology with the acetyl xylan esterase (AXE1) domain, related with acetyl xylan esterase. Signal P analysis supports prediction of secretion for protein: 514427, 526309 and 87496 and in congruence WoLF PSORT deduced that they are extracellular proteins (Table 3). 

Rapid analyses and deduction of intracellular location and large size can exclude some of these candidates, but we decided to perform further bioinformatics analyses on all these proteins in order to achieve the most effective selection of final candidates. In accordance with the search criteria, the order for extracellular TAG lipase in *T. harzianum* is 77338, 78181, 514427, 526309, 87496, 514252, 510832, 92423, 135964, 502433, 551811, 79895 and 492160.

### 3.5. Phylogenetic Analysis

**Figure 2:**
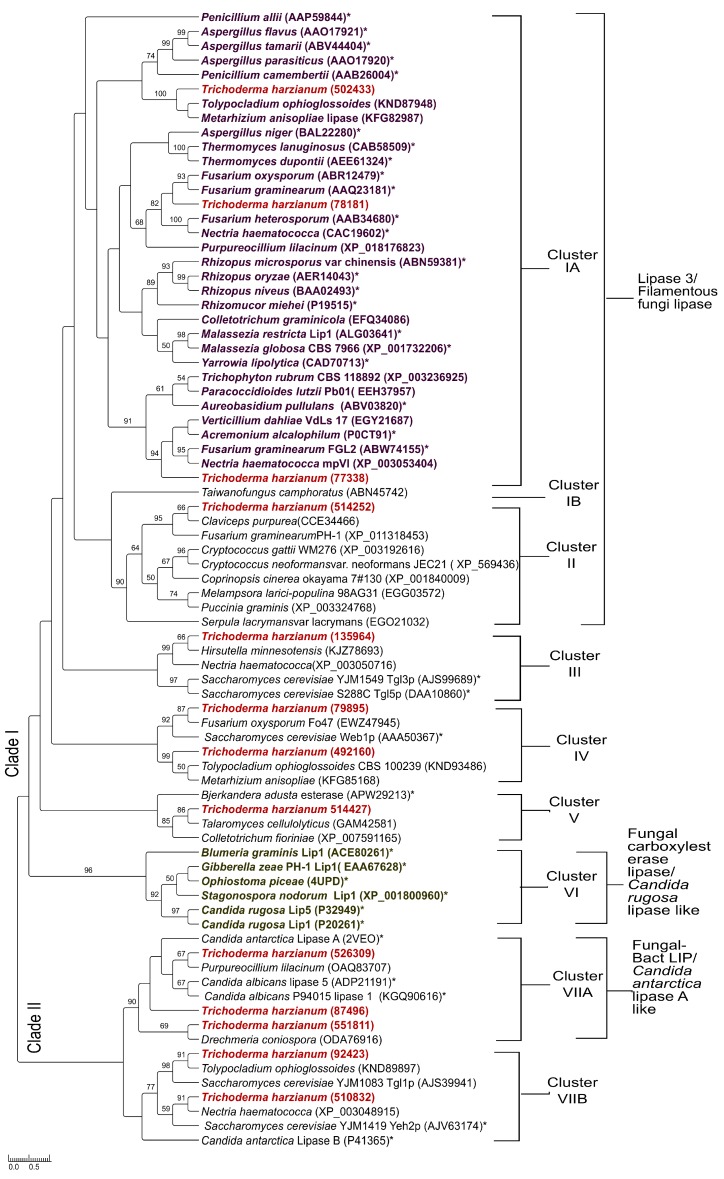
Phylogenetic tree of fungal lipases. The tree was constructed with 35 characterized fungal lipases (accessions correspond to GenBank unless another source is specified) and 15 putative triacylglycerol lipases from *T. harzianum*. (*) after the accession numbers are lipases which have been characterized; unlabeled proteins correspond to hypothetical, uncharacterized, predicted lipases. The tree was generated by MAFFT software using the neighbor-joining method [55] with 500 bootstrap re-samplings. Clusters IA, IB and II as described by Yadav et al. [6]. Highlighted in purple letters, the *Rhizomocur miehei* lipase-like group and in olive green letters, the *Candida rugosa* lipase-like group, according Schmidt-Dannert [44]. Clade I and Clade II, are consistent with Gupta et al. [76]. *T. harzianum* triacylglycerol lipases from this study, highlighted in bold red letters.

In order to find a relationship with other fungal lipases, a phylogenetic tree was constructed. We used 66 lipase sequences, 35 of which have been characterized, plus the 13 sequences of the candidates. The characterized fungal (Ascomycete and Basidiomycete) lipases/esterases were included as guides for functions. As far as we know, this is the first genome survey to retrieve true lipases in *T. harzianum* and the first phylogeny analysis including its lipases. Seven clusters were defined (Figure 2). Clusters 1A, 1B and II are largely consistent with the topology of the phylogenetic tree published by Yadav et al. [6] where filamentous Ascomycete (cluster I) and Basidiomycete (cluster II) are apart (Figure 2) but differs in the branching structure found by Feng et al. [47] and Barriuso and Martínez [75] for Lip1 type lipases and Basidiomycete lipases. The reports presented above placed Lip1-type and Basidiomycete lipases in sister clusters and here Lip1 type lipases placed in the cluster of *C. rugosa* type lipases (cluster VI), apart from Basidiomycetes. We obtained the same branching structure as those authors when a few amino acid sequences were aligned (data not shown); many phylogenetic trees of fungal lipases have been constructed focusing on a narrow group of lipases [45,47,48], which forces unrelated proteins to group together.

Our result is congruent with early phylogeny proposed by Schmidt-Dannert [44] by placing the “*Rhizomucor miehei* lipase family” clearly apart (highlighted in the phylogenetic tree in purple letters) and the “*Candida rugosa* lipase family” (highlighted in the phylogenetic tree in olive green letters and corresponds to cluster VI; see Figure 2). 

Cluster III comprises Tgl lipases and homologues; cluster IV is a branch comprising uncharacterized proteins, probably related with intracellular protein traffic; cluster V contains *B. adusta* and homologues. Cluster VII has two sub clusters where VIIA comprises *C. antarctica* lipase A and homologues. 

The topology of the phylogenetic tree reconstructed here is largely congruent with the phylogenetic tree published recently by Gupta et al. [76]. These authors carried out a comprehensive analysis on lipases available in the LED data base and then conducted the phylogenetic analysis of 61 representative lipases with different combinations of oxyanion and pentapeptide sequences in fungal and yeast lipases; their tree defined two clades. From our phylogenetic tree, the clusters I, III, V and VI belong to clade I and the cluster VII belongs to clade II. Clusters II and IV were not represented in their phylogenetic tree. Predicted proteins in cluster IV share low homology with known lipases and no member in this cluster has been characterized to date but reasons for exclusion of Basidiomycetes (cluster II) are unclear. Other differences between both phylogenetic trees is that they included 14 lipase sequences from *Y. lipolytica* (most of them placed in the clade I) and 10 lipase sequences from *C. albicans* (most of them placed in the clade II, resulting in a larger clade II in their phylogenetic tree, in comparison with the cluster II in the phylogenetic tree presented here). The most important difference is that class 3 lipases comprise a single cluster in our phylogenetic tree but these lipases split between clade I and clade II in theirs. This difference is because all *T. harzianum* class 3 lipases and their homologues, are lipases with the pentapeptide GHSLG, which is the most frequent in this class of lipases. However, class 3 lipases can contain the pentapeptides GTSAG, GHSFG and GHSYG [77]. Gupta et al. [76] included class 3 lipases with different pentapeptides. So, the differences between both phylogenetic trees are apparent but they are actually quite consistent. The consistency in the topology of our phylogenetic tree with, Yadav et al. [6], Schmidt-Dannert [44] and Gupta et al. [76] validates it and supports the phylogeny of the candidate proteins.

The four lipases class 3 place in different branches in the large cluster I of “Filamentous fungi lipase” (Figure 2). Class 3 family comprises true lipases [31,40] and they are characterized as having an active site flap/lid and work on long-chain acyl-triglycerides. In consequence, these four class 3 lipases became strong candidates. The protein 78181 placed in the same cluster as *F. graminearun* FGL1 AAQ23181, *F. heterosporum* AAB34680 and *T. lanigunosus* CAB58509. 

The protein 77338 clustered with P0CT91, LipA of *Acremonium alcalophilum*, a protein which is a lipase/acetylxylan esterase activity that works on long-chain pNP esters and xylans [50]. The protein 502433 grouped with uncharacterized lipases, in a sister clade that grouped lipases involved in aflatoxin B-producing in *Aspergillus flavus*, *Aspergillus tamari* and *Aspergillus parasiticum*. 502433 is probably related with the production of toxic metabolites against *T. harzianum* antagonists. 

*T. harzianum* 514252 grouped in the cluster II with lipases from *C. purpurea* and *F. graminearum*, in a sister clade to lipases from Basidiomycetes (*Cryptococcus gatti*, *Cryptococcus neoformans*, *Coprinus cinereus*, *Melampsora larici-populina* and *Puccinia graminis*); however functional information regarding these lipases has not been elucidated. 

Three of the four proteins automatically annotated as “secreted lipases” in the *T. harzianum* portal (526309, 551811 and 87496), clustered with the *C. antarctica* Lipase A (CALA), a TAG lipase (cluster VII). The fourth predicted secreted lipase (514427) grouped with *B. adusta* (cluster V) fungal esterase APW29213, which has no activity on long-chain glycerides [53].

The protein 135964 grouped with Tgl3p and Tgl5p (cluster III) and 92423 with Tgl1 (cluster VIIB), the major TAG lipases of the yeast *S. cerevisiae* [51]. 510832 also placed close to Tgl1.

The protein 492160 and 79895 grouped with *F. oxysporum* EWZ47945, *S. cerevisiae* Web1p AAA50367, *T. ophioglossoides* KND93486 and *M. anisopliae* KFG85168 in cluster IV. All of which are uncharacterized proteins originating from genomic sequencing projects and none predicted as lipase. 

### 3.6. Structural Modeling

Currently there are a number of modeling programs available and practically all have pros and cons. In this work Swiss model, HHpred and I-TASSER were simultaneously used for analyzing the lipase candidates. 

For most of the putative lipases under analysis, the protein database (PDB) accession for the best model coincided for two of the three types of modeler software and the third software retrieved a PBD accession which corresponded to another version of the same, or similar protein. For example, the proteins 526309, 87496, 551811 and 514427 hit PDB-3GUU in Swiss model, corresponding to *C. antarctica* Lipase A and in HHpred server retrieved top hit PDB-2VEO, which is the crystal structure of *C. antarctica* lipase A in its closed state. BLAST searching in specialized α/β-hydrolases/lipase-esterase databases was consistent to support homology-based and structure-based transfer annotation for most of the *T. harzianum* lipases revised here. The summary is presented in Table 4. 

General characteristics in lipases comprise α/β hydrolase fold, a catalytic triad (Ser-Asp/Glu-His) and the pentapeptide motif GXSXG [84]. The catalytic triad found here was Ser-Asp-His. Most of the predicted proteins under revision here have the pentapeptide GXSXG but two other versions were identified, AHSMG and CHSQG. The pentapeptide AXSXG is in the lipase family 1.4 found in many bacteria and it has been found in two yeasts, *Trichospora asahii* lipase TALipA, with activity on p-NPC-18 [85] and *S. cerevisiae* TGL2, which prefers short chain-TAG but retains 20% of relative activity on long chain C18:1 TAG [86]. CHSQG pentapeptide has not been identified in any characterized lipase so far. 

Three-dimensional superposition with IPBA software of each *T. harzianum* lipase model with its respective best template model permitted the identification of putative lid domain, catalytic triad, the pentapeptide sequence and the oxyanion hole in most of the candidate proteins. Specific position of each domain and motif in each *T. harzianum* TAG lipase is described in Table 4. 

Identity of lipase was not supported for two protein candidates: models for protein 79895 in Swiss model and HHpred were SEC3-type transport proteins, involved in the structure of the COPII coat transport-vesicles on membranes. I-TASSER identified an RNA-dependent RNA polymerase of Cypovirus. The search for homologues in ESTHER and LED data bases retrieved no hits.

The other unsupported candidate was 492160. Swiss model resulted in hits with low coverage (288 to 743 amino acids) over this large protein (which is 1782 amino acids). The first hit was PDB-1Y64 (30.38% identity), which corresponds to Bni1p formin from *S. cerevisiae*, complexed with ATP-actin. This is a calcium-dependent and ATP-dependent protein binding, involved in actin filament bundle assembly and cytoskeleton remodeling. HHpred showed homology with different proteins at N- and C-ends. At N end, the first hit was 3EG5, a Rho-Diaphanus-binding protein involved in cytoskeleton assembly and cell division. At C-end the first hit was PDB-2J1D, actin binding for actin assembly. No hit was retrieved in any of the modeler servers belonging to lipase or alpha-beta hydrolase families. I-TASSER was also unable to model 492160 with a non-lipase protein. BLASTp in ESTHER and LED data bases retrieved no homologues for this candidate. 

### 3.7. Functional Prediction of Candidates

Different strategies have been used to predict function. These strategies comprise sequence *homology* (e.g., BLAST), phylogenetic relationship, structure homology (i.e., detection of classic domains and motifs) and three-dimensional homology. Conducting a search with any one of them results in satisfactory functional prediction for proteins with large conservation with characterized proteins; however, more difficulties arise when proteins show low identity with known proteins, as in the case of lipases. To overcome this problem, function prediction was based on the sum of those function-transfer strategies. 

The proteins 77338, 78181, are related with extracellular TAG lipases. 77338 modeled with PDB-3O0d (Table 4), an olive oil- induced lipase from the non-pathogenic yeast *Y. lipolitica* [87], with barely 35% identity and it placed closer to LipA (Figure 2), a lipase/acetylxylan esterase from *A. alcalophilum* which works on long-chain pNP esters and xylans [50]. 

78181 is close to lipases involved in pathogenicity such as CAC19602 from *N. haematococca* [88], *F. heterosporum* AAB34680 [29] and AAQ23181 (FGL1) from *F. graminearium* [28] which are able to work on vegetable oils, including olive oil. The latter being identified as the best 3D model for 78181 (Table 4) and FGL1 expression is induced by olive oil [28]. 77338 and 78181 therefore are plausible candidates for what is being sought. 

Information for 514252 is not consistent. Fragments of this protein share homology with lipases class 3 and carboxyl esterases. 514252 has fragments of sequences with homology with domains of lipases involved in the disintegration of autophagic bodies inside the vacuole at starvation (Table S2) and the closest homologue (75% identity) is XP_011318453 and autophagy related lipase from *F. graminearum* [89], reinforcing an intracellular role. However, the protein 514252 is predicted extracellular, which is not congruent with the expected location of this protein in the cell. 

502433 is a large protein (115 kDa); it shares homology with class 3 lipases, it is predicted intracellular (Table 3) and probably function in relation to the binding of heat-shock proteins (Table S2). This kind of putative lipases/calmodulin binding heat shock proteins are predicted in few genomic data but so far, they have not been characterized at all. 

The proteins 514427, 526309, 87496 and 551811 share homologies with CALA, a lipase with interfacial activation. CALA is a secreted lipase from *C. antarctica* that catalyzes hydrolysis on long-chain TAGs such as triolein and olive oil [9]. For 551811 Signal P and WoLF PSORT give results that do not support its secretion (Table 3), decreasing its eligibility. The other three are predicted extracellular, as expected for the activity in the fungal B13-1 strain. Gene Ontology relates 514427 with iron ion transport and searching by PSI-BLAST found homology of this protein with acetyl xylan esterases. 

92423 and 510832 are probably related to each other. Both share homology with acidic lipases (Table 4), with cholesterol esterases and pimeloyl-ACP methyl ester carboxylesterases (Table 2). Congruent with these probable functions, both are predicted intracellular (Table 3).

135964 has a small size but seems to be related with intracellular protein transport from endoplasmic reticulum (Table S2, Table 2 and Table 3). 

Analyses of 79895 and 492160 do not support their association with lipases; only small fragments of their sequences share homology with lipases. The former is related with proteins involved in the traffic of proteins from endoplasmic reticulum to the Golgi apparatus and the latter with GTP-binding and organization of actin in cytoskeleton (Table 2). Congruent with their putative roles, both of them are predicted intracellular (Table 3).

### 3.8. Refining of Selection of Candidates 

Based on sequence analysis (protein size and prediction of cellular location), structure analysis (identification of domain and motifs), phylogenetic analysis (clustering with known true lipases or known non-true lipases), 3D-modeling and function prediction, the list of initial 13 candidates was reduced to a final list of candidates. Since the quest is for the gene/protein responsible for *T. harzianum*’s extracellular olive oil-lipolytic activity and as we hypothesized that the responsible lipase(s) is or are true lipases, the criteria for selecting the final list of candidates are: small size (<80 kDa) [73,74], predicted secreted, preferably with lid domain (which means interfacial activation, common in true lipases), preferably with homology with lipases active on long-chain acyl-triglycerides, including vegetable oils, such as olive oil. The candidates that meet these criteria are five: 77338, 78181, 514427, 526309 and 87496. It was decided to include 514252 in the list, since it shows domains of lipase class 3 (this class has interfacial activation) and it is predicted extracellular, although its function could not be proposed. However, sufficient information is not yet available to support its exclusion. 

### 3.9. RT-PCR Analysis for Validation of Predicting Results

**Figure 3:**
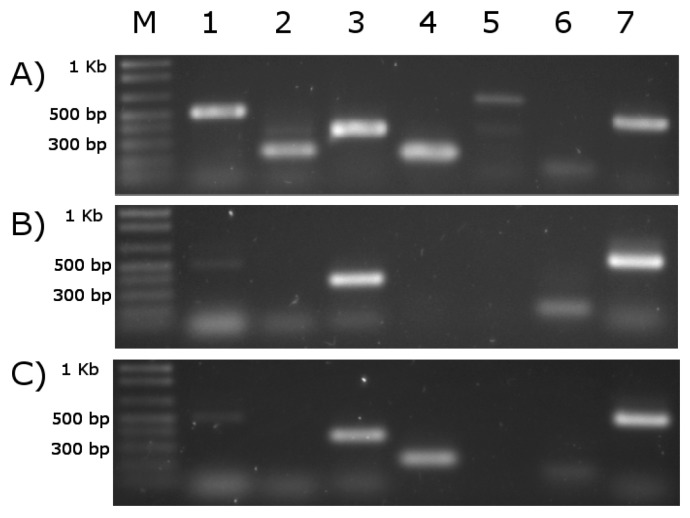
Reverse transcription polymerase chain reaction (RT-PCR) analysis of selected putative extracellular triacylglycerollipases from *Trichoderma harzianum* in medium without (**B**) or with 1% (*v*/*v*) olive oil (**C**) as carbon source. Lane (M) 1 Kb plus DNA Ladder (ThermoFisher, Carlsbad, CA, USA). The number of ID at genome portal of each candidate lipase corresponds to: (1) 77338; (2) 78181; (**3**) 514252; (4) 526309; (5) 514427; and (6) 87496. Lane (7) Elongation factor 1 (400 bp), as positive control of PCR. Panel (**A**) corresponds to PCR on genomic DNA, to test the primers. Base pair (bp).

All primer pairs, except the primer for 87496, specifically amplified the expected PCR product (Figure 3A). We were unable to screen the expression of 87496 because the primers failed to amplify even on DNA template, despite multiple attempts. 

**Figure 4:**
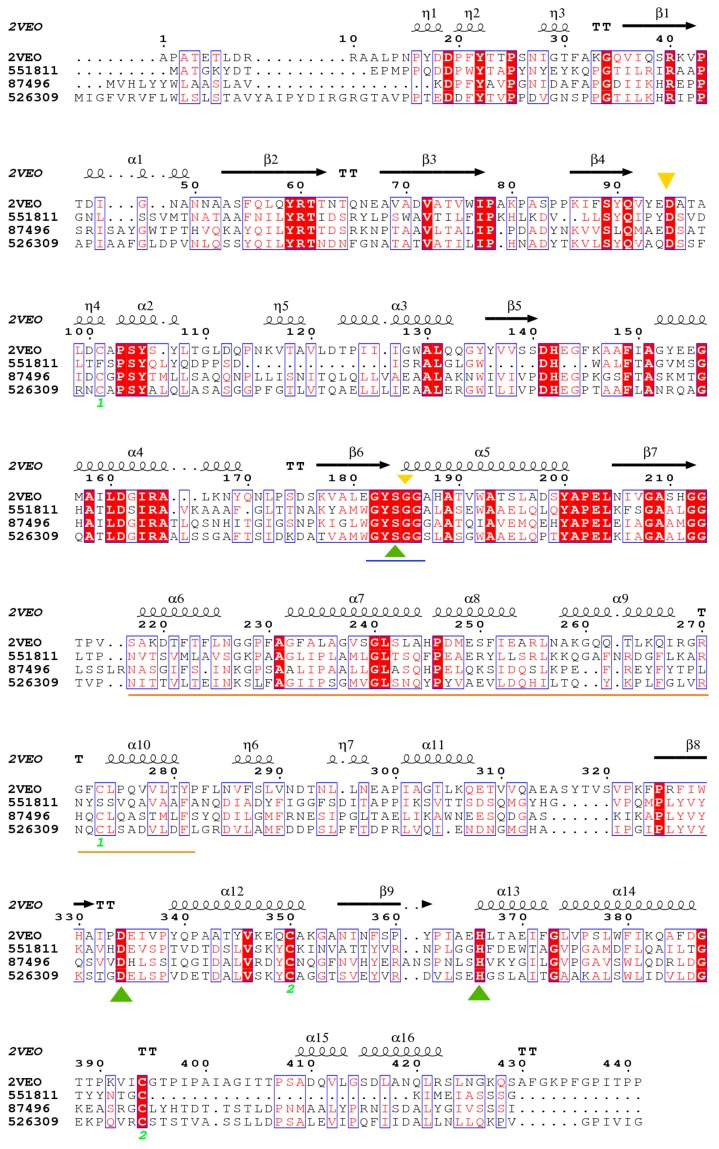
Structural-based multi-alignment of *T. harzianum* 526309, 551811 and 87496 with 2VEO lipase from *C. antarctica* (CALA). Blue line highlights the pentapeptide; orange line, the lid domain; green triangles, the catalytic triad; and yellow triangles, the oxyanion. Alpha helices and beta sheets are indicated at the top. Identical (bold white letters on red background) and similar amino acids (standard red letters) are shown.

RT-PCR analysis evidenced the olive oil-responding expression of 526309 in *T. harzianum* B13-1 strain (Figure 3C). Lipases 77338 and 514252 were also expressed but only 526309 was expressed de novo (Figure 3B,C). Thus, the RT-PCR demonstrates that the bioinformatic analyses performed here worked successfully to select candidates to explain the lipolytic activity in the strain B13-1. This result gives support to the possibility that the gene estExt_fgenesh1_pg.C_1_t10183, located at scaffold 1:494922-496430 (-) and which codifies the protein 526309, is responsible for the extracellular lipolytic activity of the *T. harzianum* B13-1 strain on olive oil. The closest homologue for 526309 is CALA, with 36% identity. This low conservation between 526309 and CALA does not discard functional relationship. Widmann et al. [90] reported 32 lipases from different sources phylogenetically related with CALA and they share a similar tridimensional structure with this protein but low identity at sequence level (as low as 15%), as found for homologues in *T. harzianum*. Conservation is usually low in lipases [41,91]. Figure 4 shows the multi-alignment of 526309 and the other two predicted secreted lipases in *T. harzianum* (this study), as well as CALA protein. They share conservation at catalytic triad and pentapeptide sequences, while lids are composed of amino acids with similar characteristics.

*T. harzianum* has different ecological niches, i.e., plant endophyte, antagonist of a wide range of plant pathogens and it is also a cosmopolitan soil-borne fungus frequently found on decaying wood. Different TAG lipases can be involved in the different ecological niches of the fungus. In the case of the lipase 526309, which is induced by olive oil, it can play role in lipid-rich environments. 

Interestingly, 526309 does not belong to class 3 of the lipases and is not even annotated as TAG lipase in the genome database of *T. harzianum* but Brennes and Baeck [92] showed that CALA, its homologue, works on long-chain TAGs. Therefore, transfer function based on structure-homology predicts triacylglycerol lipolytic activity for 526309. 

It is important to clarify that class 3 lipases are true lipases but not all true lipases belong to class 3 [93]. The most abundant amino acids in 526309 are Alanine (10.8%), Leucine (11.5%), Glycine (8.4%) and Serine (7.5%), congruent with those found in true lipases by Messaoudi et al. [93]. Three-dimensional modeling identified on 526309 the lid domain from Asn243 to Asp332 (Table 4), which is compatible with the criteria we proposed in our search. 

The survey emphasized proteins annotated in the genome database of *T. harzianum* as TAG lipases but was not restricted to them and that was a success which permitted the identification of this lipase. Cloning of the full-length cDNA of lipase 526309 is in progress in our laboratory for heterologous expression and further characterization of this protein, to challenge this proposal. 

This is the first report on the identification and in silico characterization of TAG lipases from *T. harzianum* and expands the catalogue of potential enzymes available for industrial applications. The combined strategy of functional screening of isolated microorganisms and bioinformatic analyses, if the genome sequence is available, can be applied to identify lipases in other microorganisms, or to identify other particular enzymes among a large family of protein candidates. To date, there are a large number of sequenced genomes of fungi and bacteria. 

## 4. Conclusions

In this work, we reported one *T. harzianum* strain as the best producer of extracellular lipolytic activity among the fungal strains analyzed here. A search in the deduce proteome at *T. harzianum* genomic portal retrieved 50 lipases. Bioinformatics survey managed to reduce the list to seven candidate proteins and RT-PCR analysis identified de novo expression of one of those candidates when the fungus grew on olive oil-containing medium, supporting the suitability of the procedure followed here. 526309 shares homology with CALA lipase and has potential for further application in industry.

## Figures and Tables

**Table 1:** Fungi isolated in this study from banana plant residue and used for screening of lipolytic activity.

Strain	Morphology	Extracellular Lipase at 48 h *
A04-5	Black, concentric rings	+
A06-6	White, radial growth	+
B07(+)-1(3)N	White, radial growth	-
B08-6	White, radial growth and Green spores	+
B09-4	White, cottony mycelia	-
B09-5	White, cottony mycelia	++
B09-8	White, radial growth	++
B10-4(b1)Emmb	Black, radial growth	-
B10(+)-2(4)	White, radial growth	-
B10-4(1)-2(1)	Black, radial growth	-
B11-6	Fuchsia, cottony mycelia	++
B11-7	Fuchsia, cottony mycelia	++
B13-1	White with green concentric rings	+++
B13-3	White with green concentric rings	+++
B13-4	Yellow, compact colony	++
B14-6	White, radial growth	++
B17(+)-4(3)	Fuchsia, cottony mycelia	++
B19-01-3(3)	White, radial growth	+

* Visual observation according (Carissimi et al. [15]; Ortiz-Lechuga et al. [8]). Hours (h).

**Table 2:** List of domains and family description of putative true lipases of *Trichoderma harzianum* *.

Protein ID	Superfamily	Domains	Domain Description	Active Site Domain	Substrate Binding Pocket	BLAST Hits (Search Homologues in the First 50 Hits)	PSI-BLAST (Homologues Characterized)
551811	Abhydrolase family cl21494	LIP pfam03583 Mal_quin-oxido TIGR01320	Secretory lipase: related with lipases from *Candida albicans*. Malate:quinone-oxidoreductase: Membrane-associated enzyme as part of the TCA cycle	Active site Ser-His-Asp/Glu. Nucleophilic attack on a carbonyl carbon atom.	Substrate binding pocket related to pfam03583	Genome Sequence and Annotation of different fungi (e.g., *Trichoderma* sp., *Metharhizium* sp., *Fusarium* sp.) as hypothetical protein and Predicted lipases	Lipase 1 *C. albicans* (KGR02689) Query cover 94% Identity 33%
87496	Abhydrolase family cl21494	LIP pfam03583	Secretory lipase: Related with lipases from *C. albicans*	Active site Ser-His-Asp/Glu. Nucleophilic attack on a carbonyl carbon atom.	Substrate binding pocket related to pfam03583	Genome Sequence and Annotation of different fungi (e.g., *Trichoderma* sp., *Metharhizium* sp., *Fusarium* sp.) as hypothetical protein and Predicted lipases	Lipase 1 *C. albicans* (KGR02689) Query cover 93% Identity 32%
526309	Abhydrolase family cl21494	LIP pfam03583	Secretory lipase: Related with lipases from *C. albicans*	Active site Ser-His-Asp/Glu. Nucleophilic attack on a carbonyl carbon atom.	Substrate binding pocket related to pfam03583	GH16 protein [*Trichoderma guizhouense*]. Genome Sequence and Annotation of different fungi (e.g., *Trichoderma* sp., *Metharhizium* sp., *Fusarium* sp., *Pochonia* sp., *Cordyceps* sp., etc.) as hypothetical protein. Secretory lipase. Probable lipase precursor	Secretory lipase 5 *C. albicans* (ADP21191.1) Query cover 92% Identity 39%
514427	Abhydrolase family cl21494	LIP pfam03583 DAP2	Secretory lipase; Related with lipases from *C. albicans* Dipeptidyl aminopeptidase/acylaminoacyl peptidase:Amino acid transport and metabolism Acetyl xylan esterase (AXE1).	Active site Ser-His-Asp/Glu. Nucleophilic attack on a carbonyl carbon atom.	Substrate binding pocket related to pfam03583	Genome Sequence and Annotation of different fungi (e.g., *Trichoderma* sp., *Metharhizium* sp., *Fusarium* sp.) as hypothetical protein and prolyl aminopeptidase (secreted protein).	No characterized protein among the first 500 hits
510832	Alpha/Beta hydrolase fold cl26327	PLN02872 Abhydro_lipase (pfam04083) Mhpc (COG0596)	Triacylglycerol lipase Partial alpha/beta hydrolase lipase region: Pimeloyl-ACP methyl ester carboxylesterase	-	-	Genome Sequence and Annotation of different fungi (e.g., *Trichoderma* sp., *Metharhizium* sp., *Fusarium* sp., *Colletotrichum* sp., etc.) as hypothetical protein, triglyceride lipase-cholesterol esterase, alpha/beta-hydrolase, Sterol esterase 2.	Yeh2p *Saccharomyces cerevisiae* YJM1419 (AJV63174). Query cover 58% Identity 35%
92423	Alpha/Beta hydrolase fold cl26327	PLN02872 Abhydro_lipase (pfam04083) MhpC (Cpg0596)	Triacylglycerol lipase Partial alpha/beta-hydrolase lipase region Pimeloyl-ACP methyl ester carboxylesterase	-	-	Genome Sequence and Annotation of different fungi (e.g., *Trichoderma* sp., *Metharhizium* sp., *Fusarium* sp., *Colletotrichum* sp., etc.) as steryl ester lipase TPL1, lysosomal acid lipase/cholesteryl ester hydrolase, triglyceride lipase-cholesterol esterase, hypothetical protein, alpha/beta hydrolase fold-1	Tgl1p *S. cerevisiae* YJM1083 (AJS39941) Query cover 80% Identity 43%
79895	WD40 Cl25539	WD40 (cd00200) WD40 (COG2319) WD40 (smart00320) Wd40 (pfam00400) PHA03247 Atrophin-1 (pfam03154) PLN00171 Amelogenin (smart00818) PABP-1234 (TIGR01628)	WD40 domain, found in many eukaryotic proteins with a wide variety of functions. Ancestral coatomer element 1 (ACE1) of COPII with role in vesicular traffic. Atrophin-1 family domain Protein SPA1-related Cell adhesion proteins Polyadenylate binding protein:	-	-	Genome Sequence and Annotation of different fungi (e.g., *Trichoderma* sp., *Metharhizium* sp., *Fusarium* sp., *Colletotrichum* sp., etc.) as hypothetical protein, Vesicle coat complex COPII, Sec31, transport protein sec31, transporter.	Web1p *S. cevevisiae* (AAA50367) Query cover 99% Identity 31%
135964	Abhydrolase family cl21494.	EstA (COG1075) PGAP1 (pfam07819)	Triacylglycerol esterase/lipase PGAP1-like protein	Active site Ser-His-Asp/Glu. Nucleophilic attack on a carbonyl carbon atom.	-	Genome Sequence and Annotation of different fungi (e.g., *Trichoderma* sp., *Hirsutella* sp., *Stachybotrys* sp., *Fusarium* sp., *Ophiocordyceps* sp., *Colletotrichum* sp., etc.) as hypothetical protein, Triacylglycerol lipase, Lipase 2, related to TGL2-triacylglycerol lipase, GPI inositol-deacylase PGAP1-like protein, PGAP1-like protein lipase.	No characterized protein among the first 500 hits
492160	DRF_GBD Superfamily Cl05720 DRF_FH3 Superfamily Cl05717 FH2 Superfamily cl19758	Drf_GBD (pfam06371) Drf_FH3 (pfam06367) FH2 (pfam02181) FH2 (smart00498) PHA03307	Diaphanous GTPase-binding domain; Rho proteins, leading to activation of the Drf protein. Formin Homology 2 Domain Involved in rearrangements of the actin cytoskeleton. Transcriptional regulator ICP4-like	-	-	Genome Sequence and Annotation of different fungi (e.g., *Trichoderma* sp., *Tolypocladium* sp., *Pochonia* sp., *Metharhizium* sp., *Fusarium* sp., *Colletotrichum* sp., etc.) as cytokinesis protein sepA, Rho-GTPase effector BNI1 and related formin, involved in mating, karyogamy and meiosis, hypothetical protein, SepA/Bni1	PSI-BLAST analysis did not run
77338	Abhydrolase family cl21494.	Lipase_3 (cd00519) Lipase_3 (pfam001764) PLN02310 Lip2 (COG3675)	Lipases (Class 3). Lipase that can hydrolyze long-chain acyl-triglycerides into di- and monoglycerides, glycerol and free fatty acids.	Active site Ser-His-Asp/Glu. Nucleophilic elbow on conserved domain Lipase_3: GHSLG	Active site flap/lid on conserved domain Lipase_3 (11 amino acids).	Genome Sequence and Annotation of different fungi (e.g., *Trichoderma* sp., *Tolypocladium* sp., *Pochonia* sp., *Metharhizium* sp., *Fusarium* sp., *Colletotrichum* sp., etc.) as lipase, hypothetical protein, triacylglycerol lipase, Mono- and diacylglycerol lipase, Putative feruloyl esterase A, Alpha/Beta hydrolase protein.	Triacylglycerol lipase FGL2 *Fusarium graminearum* (ABW74155) Query cover 89% Identity 62%
514252	Lipase (class 3). Alpha/beta hydrolase cd00519	Lipase_3 (pfam01764) Lipase_3 (cd00519) CVT17 (COG5153) PRK11071	Lipase that can hydrolyze long-chain acyl-triglycerides into di- and monoglycerides, glycerol and free fatty acids. Putative lipase essential for disintegration of autophagic bodies inside the vacuole Esterase YqiA	Active site Ser-His-Asp/Glu. Nucleophilic elbow on conserved domain Lipase_3: GHSLG	Active site flap/lid on conserved domain Lipase_3 (11 amino acids).	Genome Sequence and Annotation of different fungi (e.g., *Trichoderma* sp., *Tolypocladium* sp., *Pochonia* sp., *Metharhizium* sp., *Fusarium* sp., *Colletotrichum* sp., etc.) as autophagy related lipase Atg15, triacylglycerol lipase, hypothetical protein, alpha/beta-hydrolase, related to starvation induced protein PSI-7, autophagy lipase.	Hypothetical protein FGSG_02519 *F. graminearum* PH-1 (XP_011318453) Query cover 92% Identity 70%
502433	Predicted triacylglycerol lipase activity, Lipase_3.	Lipase 3 (cd00519) Lipase_3 (pfam01764) PLN02847 AF-4 (pfam05110)	Lipase that can hydrolyze long-chain acyl-triglycerides into di- and monoglycerides, glycerol and free fatty acids. AF-4 Proto-oncoprotein; Nuclear proteins linked to human disease	Active site Ser-His-Asp/Glu. Nucleophilic elbow on conserved domain Lipase_3: GHSLG	Active site flap/lid on conserved domain Lipase_3 (11 amino acids).	Genome Sequence and Annotation of different fungi (e.g., *Trichoderma* sp., *Tolypocladium* sp., *Pochonia* sp., *Metharhizium* sp., *Ophiocordyceps* sp., *Fusarium* sp., *Colletotrichum* sp., etc.) as esterase/lipase, hypothetical protein, Sn1-specific diacylglycerol lipase beta, Lipase, Lipase, class 3,	No characterized protein among the first 500 hits
78181	Predicted triacylglycerol lipase activity, Lipase_3. Lipid transport and metabolism.	Lipase_3 (cd00519) Lipase_3 (pfam01764) PLN00413 Lip2	Lipase that can hydrolyze long-chain acyl-triglycerides into di- and monoglycerides, glycerol and free fatty acids.	Active site Ser-His-Asp/Glu. Nucleophilic elbow on conserved domain Lipase_3: GHSLG	Active site flap/lid on conserved domain Lipase_3 (11 amino acids).	Genome Sequence and Annotation of different fungi (e.g., *Trichoderma* sp., *Purpureocillium* sp., *Fusarium* sp., *Tolypocladium* sp., etc.) as extracellular lipase-like protein, hypothetical protein, Lipase, probable triacylglycerol lipase precursor, Chain A, Crystal Structure of Lipase	Lipase *Fusarium heterosporum* (AAB34680) Query cover 98% Identity 52%

* Annotation is summary of information retrieved from Gene Ontology, Conserved Domain tool at NCBI, Pfam and Superfamily.

**Table 3:** In silico analysis of putative true lipases of *Trichoderma harzianum*.

Protein ID	aa Residues	Molecular Weight (kDa)	SignalP (Secreted)	TMHMM Domains	Putative Cellular Localization	Continue for Next Analysis
551811	379	41.185	No	No	Cytoplasmic	Yes
87496	430	46.62	Yes	No	Extracellular	Yes
526309	452	47.99	Yes	No	Extracellular	Yes
514427	454	49.25	Yes	No	Extracellular	Yes
510832	722	82.80	No	No	Cytoplasmic	No
92423	552	62.361	No	1	Ambiguous location (plasmatic, endoplasmic reticulum, Golgi Apparatus)	Yes
79895	1254	134.85	No	No	Mitochondria	No
135964	339	37.51	No	No	Endoplasmic reticulum	no
492160	1782	198.07	No	No	Nuclear	No
77338	404	44.5	Yes	1	Extracellular	Yes
514252	613	65.4	Yes	No	Extracellular	Yes
502433	1059	115.6	No	No	Cytosolic/nuclear	Yes
78181	340	36.2	Yes	No	Extracellular	Yes

kDa: kilodalton; aa: amino acids.

**Table 4:** In silico localization of characteristic lipase domains in the candidate lipase proteins from *Trichoderma harzianum*.

Protein ^&^	Template	Reference Art	Pentapeptide	Lid Domain	Catalytic Triad	Oxyanion
**551811**	3GUU	Ericsson et al. [78]	GYSGG Gly160-Gly164	Asn195-Ser287	Ser162, Asp309, His341	Asp92 Gly163
**87496**			GYSGG Gly188-Gly192	Leu223-Glu313	Ser190, Asp334, His368	Asp96 Gly191
**526309**			GYSGG Gy208-Gly212	Asn243-Asp332	Ser210, Asp352, His384	Asp116 Gly211
**514427**			GHSQG Gly226-Gly230	Ala258-Phe349	Ser228, Asp373, His405	Ile146 Gln229
**510832**	1K8Q	Roussel et al. [79] and Selvan et al. [80]	CHSQG Cys431-Gly435	Phe496-Glu550	Ser433, Asp634, His667	Leu349 Gln434
**92423**			GFSQG Gly224-Gly228	Ile286-Ile321	Ser226, Asp396, His422	Leu139 Gln227
**79895 ^#^**	No structural homologue of lipase was identified					
**135964**	4 X6U	Dror et al. [81]	AHSMG Ala-152-Gly156	Phe189-Pro200	Ser154, Asp275, His297	Leu70 Met155
**492160 ^#^**	No structural homologue of lipase was identified					
**77338**	3O0D	Bordes et al. [82]	GHSLG Gly214-Gly218	Thr137-Tyr154	Ser216, Asp282, His343	Thr137 Leu217
**514252**			GHSLG Gly316-Gly320	Thr230-Trp262	Ser318, Asp379, His457	Thr230 Leu319
502433 *	Complete protein sequence does not model with lipase					
**78181**	3NGM	Lou et al. [83]	GHSLG Gly174-Gly178	Asn115-Phe126	Ser176, Asp230, His289	Ser114 Leu177

* Lipase domains restricted to a short fragment of the protein. ^#^ No modeling with lipases. ^&^ Three-dimensional models are available as Figure S2.

## Supplementary Materials

The following are available online at http://www.mdpi.com/2073-4425/9/2/62/s1. Table S1: List of primers used in this study, Table S2: List of all lipase proteins identified in the *T. harzianum* genome homepage, Figure S1: Fungal strains B13-1 (A) and B13-3 (B) selected in screening for extracellular lipolytic activity using olive oil as carbon source in Rhodamine B test, Figure S2: Three-dimensional model proposed for (A) 551811; (B) 87496; (C) 526309; (D) 514427; (E) 510832; (F) 92423; (G) 135964; (H) 77338; (I) 514252; (J) 78181. In all protein models: (1) Red, α-helixes; yellow, β-strands. (2) Close up showing the lid (yellow), the catalytic triad (in red) and the oxyanion (in blue). (3) Superposition with best template protein model (see Table 4). (4) Superposition of catalytic triad in both proteins. All models were generated by I-TASSER and visualizations were performed in PyMOL.

## References

[1] Gopinath S.C.B., Anbu P., Lakshmipriya T., Hilda A. (2013). Strategies to characterize fungal lipases for applications in medicine and dairy industry. BioMed Res. Int..

[2] Verger R. (1997). ‘Interfacial activation’ of lipases: Facts and artifacts. Trends Biotechnol..

[3] Nwuche C.O., Ogbonna J.C. (2011). Isolation of lipase producing fungi from palm oil Mill effluent (POME) dump sites at Nsukka. Braz. Arch. Biol. Technol..

[4] Daiha K., Angeli R., de Oliveira S.D., Almeida R.V. (2015). Are Lipases Still Important Biocatalysts? A Study of Scientific Publications and Patents for Technological Forecasting. PLoS ONE.

[5] Andualema B., Gessesse A. (2012). Microbial lipases and their industrial applications. Biotechnology.

[6] Yadav S., Dubey A.K., Yadav S., Bisht D., Singh-Darmwal N., Yadav D. (2012). Amino acid sequences based phylogenetic and motif assessment of lipases from different organisms. Online J. Bioinform..

[7] Lanka S., Latha J.N.L. (2015). Purification and characterization of a new cold active lipase, EnL A from *Emericella nidulans* NFCCI 3643. Afr. J. Biotechnol..

[8] Ortiz-Lechuga E.G., Quintero-Zapata I., Niño K.A. (2016). Detection of extracellular enzymatic activity in microorganisms isolated from waste vegetable oil contaminated soil using plate methodologies. Afr. J. Biotechnol..

[9] Coradi G.V., da Visitação V.L., de Lima E.A., Saito L.Y.T., Palmieri D.A., Takita M.A., Neto P.O., de Lima V.M.G. (2013). Comparing submerged and solid-state fermentation of agro-industrial residues for the production and characterization of lipase by *Trichoderma harzianum*. Ann. Microbiol..

[10] Falony G., Armas J.C., Mendoza J.C.D., Hernández J.L.M. (2006). Production of extracellular lipase from *Aspergillus niger* by solid-state fermentation. Food Technol. Biotechnol..

[11] Lima V.M.G., Krieger N., Sarquis M.I.M., Mitchell D.A., Ramos L.P., Fontana J.D. (2003). Effect of Nitrogen and Carbon Sources on Lipase Production by *Penicillium aurantiogriseum*. FTB J..

[12] Ülker S., Özel A., Colak A., Karaoğlu Ş.A. (2011). Isolation, production, and characterization of an extracellular lipase from *Trichoderma harzianum* isolated from soil. Turk. J. Biol..

[13] Mier T., Toriello C., Ulloa M. (2002). Hongos Microscópicos Saprobios y Parásitos: Métodos de Laboratorio.

[14] Panuthai T., Sihanonth P., Piapukiew J., Sooksai S., Sangvanich P., Karnchanatat A. (2012). An extracellular lipase from the endophytic fungi *Fusarium oxysporum* isolated from the Thai medicinal plant, *Croton oblongifolius* Roxb. Afr. J. Microbiol. Res..

[15] Carissimi M., de Souza T.F., Corbellini V.A., Scroferneker M.L. (2007). Comparison of lipolytic activity of *Sporothrix schenckii* strains utilizing olive oil-rhodamine B and Tween 80. Tecno-Lógica.

[16] Maia M.d.M.D., Morais M.M.C.d., Morais M.A.d., Melo E.H.M., de Lima Filho J.L. (1999). Production of extracellular lipase by the phytopathogenic fungus *Fusarium solani* FS1. Rev. Microbiol..

[17] Winkler U.K., Stuckmann M. (1979). Glycogen, hyaluronate, and some other polysaccharides greatly enhance the formation of exolipase by *Serratia marcescens*. J. Bacteriol..

[18] Pacheco S., Júnior A., Morgado A., Júnior A., Amadi O., Guisán J., Pessela B. (2015). Isolation and Screening of Filamentous Fungi Producing Extracellular Lipase with Potential in Biodiesel Production. Adv. Enzyme Res..

[19] Johanson A., Jeger M.J. (1993). Use of PCR for detection of *Mycosphaerella fijiensis* and *M. musicola*, the causal agents of Sigatoka leaf spots in banana and plantain. Mycol. Res..

[20] White T.J., Bruns T., Lee S., Taylor J.L. (1990). Amplification and direct sequencing of fungal ribosomal RNA genes for phylogenetics. PCR Protoc. Guide Methods Appl..

[21] Hall T. (1999). BioEdit: A user-friendly biological sequence alignment editor and analysis program for Windows 95/98/NT. Nucleic Acids Symp. Ser..

[22] Okonechnikov K., Golosova O., Fursov M. (2012). Unipro UGENE: A unified bioinformatics toolkit. Bioinformatics.

[23] Edgar R.C. (2004). MUSCLE: Multiple sequence alignment with high accuracy and high throughput. Nucleic Acids Res..

[24] Altschul S.F., Gish W., Miller W., Myers E.W., Lipman D.J. (1990). Basic local alignment search tool. J. Mol. Biol..

[25] NCBI Resource Coordinators (2016). Database resources of the National Center for Biotechnology Information. Nucleic Acids Res..

[26] Druzhinina I.S., Kopchinskiy A.G., Komoń M., Bissett J., Szakacs G., Kubicek C.P. (2005). An oligonucleotide barcode for species identification in *Trichoderma* and *Hypocrea*. Fungal Genet. Biol..

[27] Nordberg H., Cantor M., Dusheyko S., Hua S., Poliakov A., Shabalov I., Smirnova T., Grigoriev I.V., Dubchak I. (2014). The genome portal of the Department of Energy Joint Genome Institute: 2014 updates. Nucleic Acids Res..

[28] Voigt C.A., Schäfer W., Salomon S. (2005). A secreted lipase of *Fusarium graminearum* is a virulence factor required for infection of cereals: Lipase as a virulence factor. Plant J..

[29] Nagao T., Shimada Y., Sugihara A., Tominaga Y. (1994). Cloning and Nucleotide Sequence of cDNA Encoding a Lipase from *Fusarium heterosporum*. J. Biochem..

[30] Haraldsson G.G., Kristinsson B. (2016). Process for Separating Polyunsaturated Fatty Acids from Long Chain Unsaturated or Less Saturated Fatty Acids. U.S. Patent.

[31] Marchler-Bauer A., Bo Y., Han L., He J., Lanczycki C.J., Lu S., Chitsaz F., Derbyshire M.K., Geer R.C., Gonzales N.-R. (2017). CDD/SPARCLE: Functional classification of proteins via subfamily domain architectures. Nucleic Acids Res..

[32] Finn R.D., Coggill P., Eberhardt R.Y., Eddy S.R., Mistry J., Mitchell A.L., Potter S.C., Punta M., Qureshi M., Sangrador-Vegas A. (2016). The Pfam protein families database: Towards a more sustainable future. Nucleic Acids Res..

[33] Alva V., Nam S.Z., Söding J., Lupas A.N. (2016). The MPI bioinformatics Toolkit as an integrative platform for advanced protein sequence and structure analysis. Nucleic Acids Res..

[34] Finn R.D., Attwood T.K., Babbitt P.C., Bateman A., Bork P., Bridge A.J., Chang H.-Y., Dosztányi Z., El-Gebali S., Fraser M. (2017). InterPro in 2017—beyond protein family and domain annotations. Nucleic Acids Res..

[35] Petersen T.N., Brunak S., von Heijne G., Nielsen H. (2011). SignalP 4.0: Discriminating signal peptides from transmembrane regions. Nat. Methods.

[36] Krogh A., Larsson B., von Heijne G., Sonnhammer E.L. (2001). Predicting transmembrane protein topology with a hidden Markov model: Application to complete genomes. J. Mol. Biol..

[37] Horton P., Park K.-J., Obayashi T., Fujita N., Harada H., Adams-Collier C.J., Nakai K. (2007). WoLF PSORT: Protein localization predictor. Nucleic Acids Res..

[38] Protein Molecular Weight Calculator. http://www.sciencegateway.org/tools/proteinmw.htm.

[39] Gasteiger E., Hoogland C., Gattiker A., Duvaud S., Wilkins M.R., Appel R.D., Bairoch A., Walker J.M. (2005). Protein Identification and Analysis Tools on the ExPASy Server. The Proteomics Protocols Handbook.

[40] Marchler-Bauer A., Derbyshire M.K., Gonzales N.R., Lu S., Chitsaz F., Geer L.Y., Geer R.C., He J., Gwadz M., Hurwitz D.I. (2015). CDD: NCBI’s conserved domain database. Nucleic Acids Res..

[41] Fischer M., Pleiss J. (2003). The Lipase Engineering Database: A navigation and analysis tool for protein families. Nucleic Acids Res..

[42] Hotelier T., Renault L., Cousin X., Negre V., Marchot P., Chatonnet A. (2004). ESTHER, the database of the α/β-hydrolase fold superfamily of proteins. Nucleic Acids Res..

[43] Altschul S.F., Madden T.L., Schäffer A.A., Zhang J., Zhang Z., Miller W., Lipman D.J. (1997). Gapped BLAST and PSI-BLAST: A new generation of protein database search programs. Nucleic Acids Res..

[44] Schmidt-Dannert C. (1999). Recombinant microbial lipases for biotechnological applications. Bioorg. Med. Chem..

[45] Feng J., Guosheng L., Gopalan S., Geoffrey R., Wei Y. (2005). A secreted lipase encoded by *LIP1* is necessary for efficient use of saturated triglyceride lipids in *Fusarium graminearum*. Microbiology.

[46] Feng J., Wang F., Liu G., Greenshields D., Shen W., Kaminskyj S., Hughes G.R., Peng Y., Selvaraj G., Zou J. (2009). Analysis of a *Blumeria graminis*-secreted lipase reveals the importance of host epicuticular wax components for fungal adhesion and development. Mol. Plant-Microbe Interact. MPMI.

[47] Feng J., Hwang R., Hwang S.-F., Gaudet D., Strelkov S.E. (2011). Molecular characterization of a *Stagonospora nodorum* lipase gene *LIP1*: A triglyceride lipase from *Stagonospora nodorum*. Plant Pathol..

[48] Park M., Jung W.H., Han S.H., Lee Y.H., Lee Y.W. (2015). Characterisation and Expression Analysis of MrLip1, a Class 3 Family Lipase of *Malassezia restricta*. Mycoses.

[49] Xu J., Saunders C.W., Hu P., Grant R.A., Boekhout T., Kuramae E.E., Kronstad J.W., DeAngelis Y.M., Reeder N.L., Johnstone K.R. (2007). Dandruff-associated *Malassezia* genomes reveal convergent and divergent virulence traits shared with plant and human fungal pathogens. Proc. Natl. Acad. Sci. USA.

[50] Pereira E., Tsang A., McAllister T.A., Menassa R. (2013). The production and characterization of a new active lipase from *Acremonium alcalophilum* using a plant bioreactor. Biotechnol. Biofuels.

[51] Klein I., Klug L., Schmidt C., Zandl M., Korber M., Daum G., Athenstaedt K. (2016). Regulation of the yeast triacylglycerol lipases Tgl4p and Tgl5p by the presence/absence of nonpolar lipids. Mol. Biol. Cell.

[52] Cedillo V., Plou F.J., Martínez M. (2012). Recombinant sterol esterase from *Ophiostoma piceae*: An improved biocatalyst expressed in *Pichia pastoris*. Microb. Cell Factories.

[53] Sánchez-Carbente M.R., Batista-García R.A., Sánchez-Reyes A., Escudero-Garcia A., Morales-Herrera C., Cuervo-Soto L.I., French-Pacheco L., Fernández-Silva A., Amero C., Castillo E. (2017). The first description of a hormone-sensitive lipase from a basidiomycete: Structural insights and biochemical characterization revealed *Bjerkandera adusta* Ba EstB as a novel esterase. MicrobiologyOpen.

[54] Katoh K., Toh H. (2008). Recent developments in the MAFFT multiple sequence alignment program. Brief. Bioinform..

[55] Saitou N., Nei M. (1987). The neighbor-joining method: A new method for reconstructing phylogenetic trees. Mol. Biol. Evol..

[56] Biasini M., Bienert S., Waterhouse A., Arnold K., Studer G., Schmidt T., Kiefer F., Cassarino T.G., Bertoni M., Bordoli L. (2014). SWISS-MODEL: Modelling protein tertiary and quaternary structure using evolutionary information. Nucleic Acids Res..

[57] Söding J., Biegert A., Lupas A.N. (2005). The HHpred interactive server for protein homology detection and structure prediction. Nucleic Acids Res..

[58] Yang J., Yan R., Roy A., Xu D., Poisson J., Zhang Y. (2015). The I-TASSER Suite: Protein structure and function prediction. Nat. Methods.

[59] Gelly J.-C., Joseph A.P., Srinivasan N., de Brevern A.G. (2011). iPBA: A tool for protein structure comparison using sequence alignment strategies. Nucleic Acids Res..

[60] DeLano W.L. (2002). The PyMol Molecular Graphics System.

[61] Thompson J.D., Higgins D.G., Gibson T.J. (1994). CLUSTAL W: Improving the sensitivity of progressive multiple sequence alignment through sequence weighting, position-specific gap penalties and weight matrix choice. Nucleic Acids Res..

[62] Robert X., Gouet P. (2014). Deciphering key features in protein structures with the new ENDscript server. Nucleic Acids Res..

[63] PrimerQuest Tool. https://www.idtdna.com/Primerquest/Home/Index.

[64] Rifaat H.M., El-Mahalawy A.A., El-Menofy H.A., Donia S.A. (2010). Production, optimization and partial purification of lipase from *Fusarium oxysporum*. J. Appl. Sci. Environ. Sanit..

[65] Savitha J., Srividya S., Jagat R., Payal P., Priyanki S., Rashmi G.W., Roshini K.T., Shantala Y.M. (2007). Identification of potential fungal strain (s) for the production of inducible, extracellular and alkalophilic lipase. Afr. J. Biotechnol..

[66] Winayanuwattikun P., Kaewpiboon C., Piriyakananon K., Chulalaksananukul W., Yongvanich T., Svasti J. (2011). Immobilized lipase from potential lipolytic microbes for catalyzing biodiesel production using palm oil as feedstock. Afr. J. Biotechnol..

[67] Jayaprakash A., Ebenezer P. (2010). Investigation on Extracellular Lipase Production by *Aspergillus japonicus* Isolated from the Paper Nest of *Ropalidia marginata*. Indian J. Sci. Technol..

[68] Peil G.H.S., Kuss A.V., Rave A.F.G., Villarreal J.P.V., Hernandes Y.M.L., Nascente P.S. (2016). Bioprospecting of lipolytic microorganisms obtained from industrial effluents. An. Acad. Bras. Ciênc..

[69] Rabbani M., Shafiee F., Shayegh Z., Sadeghi H.M.M., Shariat Z.S., Etemadifar Z., Moazen F. (2015). Isolation and Characterization of a New Thermoalkalophilic Lipase from Soil Bacteria. Iran. J. Pharm. Res. IJPR.

[70] Shafei M.S., Allam R.F. (2010). Production and immobilization of partially purified lipase from *Penicillium chrysogenum*. Malays. J. Microbiol..

[71] Hiol A., Jonzo M.D., Rugani N., Druet D., Sarda L., Comeau L.C. (2000). Purification and characterization of an extracellular lipase from a thermophilic *Rhizopus oryzae* strain isolated from palm fruit. Enzyme Microb. Technol..

[72] Kantak J.B., Bagade A.V., Mahajan S.A., Pawar S.P., Shouche Y.S., Prabhune A.A. (2011). Isolation, Identification and Optimization of a New Extracellular Lipase Producing Strain of *Rhizopus* sp.. Appl. Biochem. Biotechnol..

[73] Mehta A., Kumar-Saun N., Gupta R. (2016). Purification and Characterization of Lipase from thermophilic *Geobacillus* sp.. Curr. Biotechnol..

[74] Sharma D., Sharma B., Shukla A.K. (2011). Biotechnological approach of microbial lipase: A review. Biotechnology.

[75] Barriuso J., Martínez M.J. (2017). Evolutionary history of versatile-lipases from Agaricales through reconstruction of ancestral structures. BMC Genom..

[76] Gupta R., Kumari A., Syal P., Singh Y. (2015). Molecular and functional diversity of yeast and fungal lipases: Their role in biotechnology and cellular physiology. Prog. Lipid Res..

[77] Zan X., Tang X., Chu L., Zhao L., Chen H., Chen Y.Q., Chen W., Song Y. (2016). Lipase genes in *Mucor circinelloides*: Identification, sub-cellular location, phylogenetic analysis and expression profiling during growth and lipid accumulation. J. Ind. Microbiol. Biotechnol..

[78] Ericsson D.J., Kasrayan A., Johansson P., Bergfors T., Sandström A.G., Bäckvall J.-E., Mowbray S.L. (2008). X-ray Structure of *Candida antarctica* Lipase A Shows a Novel Lid Structure and a Likely Mode of Interfacial Activation. J. Mol. Biol..

[79] Roussel A., Miled N., Berti-Dupuis L., Rivière M., Spinelli S., Berna P., Gruber V., Verger R., Cambillau C. (2002). Crystal Structure of the Open Form of Dog Gastric Lipase in Complex with a Phosphonate Inhibitor. J. Biol. Chem..

[80] Selvan A., Seniya C., Chandrasekaran S.N., Siddharth N., Anishetty S., Pennathur G. (2010). Molecular dynamics simulations of human and dog gastric lipases: Insights into domain movements. FEBS Lett..

[81] Dror A., Kanteev M., Kagan I., Gihaz S., Shahar A., Fishman A. (2015). Structural insights into methanol-stable variants of lipase T6 from *Geobacillus stearothermophilus*. Appl. Microbiol. Biotechnol..

[82] Bordes F., Barbe S., Escalier P., Mourey L., André I., Marty A., Tranier S. (2010). Exploring the Conformational States and Rearrangements of *Yarrowia lipolytica* Lipase. Biophys. J..

[83] Lou Z., Li M., Sun Y., Liu Y., Liu Z., Wu W., Rao Z. (2010). Crystal structure of a secreted lipase from *Gibberella zeae* reveals a novel “double-lock” mechanism. Protein Cell.

[84] Casas-Godoy L., Duquesne S., Bordes F., Sandoval G., Marty A. (2012). Lipases: An Overview. Lipases and Phospholipases.

[85] Kumari A., Gupta R. (2015). Functional Characterisation of Novel Enantioselective Lipase TALipA from *Trichosporon asahii* MSR54: Sequence Comparison Revealed New Signature Sequence AXSXG Among Yeast Lipases. Appl. Biochem. Biotechnol..

[86] Ham H.J., Rho H.J., Shin S.K., Yoon H.-J. (2010). The TGL2 gene of *Saccharomyces cerevisiae* encodes an active acylglycerol lipase located in the mitochondria. J. Biol. Chem..

[87] Najjar A., Robert S., Guérin C., Violet-Asther M., Carrière F. (2011). Quantitative study of lipase secretion, extracellular lipolysis, and lipid storage in the yeast *Yarrowia lipolytica* grown in the presence of olive oil: Analogies with lipolysis in humans. Appl. Microbiol. Biotechnol..

[88] Eddine A.N., Hannemann F., Schafer W. (2001). Cloning and expression analysis of *NhL1*, a gene encoding an extracellular lipase from the fungal pea pathogen *Nectria haematococca* MP VI (*Fusarium solani* f. sp. pisi) that is expressed in planta. Mol. Genet. Genom..

[89] Nguyen L.N., Bormann J., Le G.T.T., Stärkel C., Olsson S., Nosanchuk J.D., Giese H., Schäfer W. (2011). Autophagy-related lipase FgATG15 of *Fusarium graminearum* is important for lipid turnover and plant infection. Fungal Genet. Biol..

[90] Widmann M., Juhl P.B., Pleiss J. (2010). Structural classification by the Lipase Engineering Database: A case study of *Candida antarctica* lipase A. BMC Genom..

[91] Jaeger K.E., Ransac S., Dijkstra B.W., Colson C., van Heuvel M., Misset O. (1994). Bacterial lipases. FEMS Microbiol. Rev..

[92] Brenneis R., Baeck B. (2012). Esterification of fatty acids using *Candida antarctica* lipase A in water-abundant systems. Biotechnol. Lett..

[93] Messaoudi A., Belguith H., Ghram I., Hamida J.B. (2011). LIPABASE: A database for “true” lipase family enzymes. Int. J. Bioinform. Res. Appl..

