# Supplementary material for: Fungal Screening on Olive Oil for Extracellular Triacylglycerol Lipases: Selection of a Trichoderma harzianum Strain and Genome Wide Search for the Genes
Source: Genes (Basel). 2018 Jan 25;9(2):62. doi: 10.3390/genes9020062 (PMC5852558; doi:10.3390/genes9020062)
Supplement: Supplementary file 1 [file genes-09-00062-s001.docx]

Supplementary Material for Fungal Screening on Olive Oil for Extracellular Triacylglycerol Lipases: Selection of a *Trichoderma harzianum* Strain and Genome Wide Search for the Genes

Miguel Angel Canseco-Pérez ^1^, Genny Margarita Castillo-Avila ^1,2^, Bartolomé Chi-Manzanero ^1^, Ignacio Islas-Flores ^3^, Max M. Apolinar-Hernández ^1^, Gerardo Rivera-Muñoz ^4^, Marcela Gamboa-Angulo ^1^, Felipe Sanchez-Teyer ^1^, Yeny Couoh-Uicab ^5^ and Blondy Canto-Canché ^1,^*

**Table S1**. List of primers used in this study

| Protein ID in *T. harzianum*  database | Model name | Forward and Reverse Primers | Sequences^a^ | Size of the PCR^b^ fragment (bp) ^c^ |
| --- | --- | --- | --- | --- |
| 77338 | e_gw1.3.2527.1 | 77338F | CCAGATCACGGAGGAGAATTAC | 445 |
|  |  | 77338R | GGGCAGACTTGGAGATGAATA |  |
| 78181 | e_gw1.3.156.1 | 78181F | CTCCGCAACTTCATCACAGA | 241 |
|  |  | 78181R | CAATGGCGAAACCCTGAGTA |  |
| 514252 | fgenesh1_kg.36_#_14_#_Locus1356v1rpkm114.02 | 514252F | CACTGGCTATGAGTGTGTCTATG | 387 |
|  |  | 514252R | GGTGTCGTCGTCTTGTCTTT |  |
| 526309 | estExt_fgenesh1_pg.C_1_t10183 | 526309F | ATCCCACACAACGCAGACTA | 239 |
|  |  | 526309R | GCTTGCCGATTAGCCAGAAA |  |
| 514427 | fgenesh1_kg.83_#_1_#_Locus825v1rpkm186.18 | 514427F | GAGTTGCCGTAGCTCCTAGT | 277 |
|  |  | 514427R | ATGGTTCGGGAGGTCAAAGT |  |
| 87496 | e_gw1.7.1721.1 | 87496F | CCTCTCTCGCCGTTAAGGAT | 177 |
|  |  | 87496R | TCTTCCGGCTATCTGTCGTC |  |
| Elongation factor | estExt_Genemark1.C_1_t10440 | EF1-983F | GCY CCY GGH CAY CGT GAY TTY AT | 400 |
|  |  | EF1-1567R | ACH GTR CCR ATA CCA CCR ATC TT |  |

a: Degenerate codes: H= A, C, or T, R = A or G, Y = C or T; bPCR: Polymerase Chain Reaction; cbp: Base pair

**Table S2.** List of all lipase proteins identified in the *T. harzianum* genome homepage

| **Protein Id** | **Model name** | **Chromosome location** | **Group** | **Annotations** |
| --- | --- | --- | --- | --- |
| 510832 | fgenesh1_kg.11_#_179_#_Locus5819v1rpkm11.05 | scaffold_11:472451-475242 (-) | Triacylglycerol lipase | Triglyceride lipase-cholesterol esterase |
| 79895 | e_gw1.4.2035.1 | scaffold_4:1760760-1764647 (+) | Triacylglycerol lipase | Vesicle coat complex COPII, subunit SEC31 |
| 21954 | estExt_Genemark1.C_420014 | scaffold_42:42833-44151 (-) | Acylglycerol lipase | Lysophospholipase |
| 155645 | E0stExt_Genewise1.C_250246 | scaffold_25:244434-245914 (-) | Acylglycerol lipase | Lysophospholipase |
| 92423 | e_gw1.11.791.1 | scaffold_11:316020-317968 (+) | Triacylglycerol lipase | Triglyceride lipase-cholesterol esterase |
| 551811 | MIX15484_2_17 | scaffold_45:15003-16167 (+) | Lipase_secreted | Secretory lipase |
| 87496 | e_gw1.7.1721.1 | scaffold_7:463722-465074 (-) | Lipase_secreted | Secretory lipase |
| 77338 | e_gw1.3.2527.1 | scaffold_3:1045025-1046239 (-) | Triacylglycerol lipase | Lipase (class 3) |
| 514252 | fgenesh1_kg.36_#_14_#_Locus1356v1rpkm114.02 | scaffold_36:53708-56143 (+) | Triacylglycerol lipase | Lipase (class 3),  Putative lipase essential for disintegration of autophagic bodies inside the vacuole |
| 511179 | fgenesh1_kg.12_#_53_#_Locus7951v1rpkm3.2 | scaffold_12:152256-153471 (+) | Acylglycerol lipase | Lysophospholipase |
| 526309 | estExt_fgenesh1_pg.C_1_t10183 | scaffold_1:494922-496430 (-) | Lipase_secreted | Secretory lipase |
| 135964 | estExt_Genewise1.C_3_t10273 | scaffold_3:330453-331832 (-) | Triacylglycerol lipase | Intrinsic component of endoplasmic reticulum membrane; intracellular protein transport |
| 492160 | fgenesh1_pm.3_#_685 | scaffold_3:1875111-1880526 (-) | Triacylglycerol lipase | Rho GTPase binding, actin cytoskeleton organization |
| 78181 | e_gw1.3.156.1 | scaffold_3:1634109-1635186 (-) | Triglyceride lipase | Lipase (class 3) |
| 502433 | fgenesh1_kg.1_#_333_#_Locus3731v1rpkm31.19 | scaffold_1:1178847-1182507 (-) | Triglyceride lipase | Lipase (class 3)  Predicted lipase/calmodulin-binding heat-shock protein |
| 1117 | gm1.1117_g | scaffold_1:3774719-3775867 (+) | Alpha/beta hydrolase | Hormone-sensitive lipase HSL |
| 545835 | MIX9508_25_78 | scaffold_21:72679-74243 (+) | Lipase_GDXG_HIS_AS, AB_hydrolase | Arylacetamide deacetylase |
| 95431 | e_gw1.14.395.1 | scaffold_14:945848-948060 (-) | Alpha/beta hydrolase | Hormone-sensitive lipase HSL |
| 183965 | CE14116_52606 | scaffold_1:1725128-1726866 (+) | Alpha/beta hydrolase | Hormone-sensitive lipase HSL |
| 503030 | fgenesh1_kg.1_#_930_#_Locus4686v1rpkm20.07 | scaffold_1:2789190-2793166 (+) | Predicted alpha/beta hydrolase | DUF676_lipase-like |
| 525479 | estExt_fgenesh1_pm.C_260025 | scaffold_26:94511-96195 (-) | GDSL-like Lipase/Acylhydrolase | Hydrolase activity on ester bonds |
| 554838 | MIX18511_21_34 | scaffold_7:437392-440685 (-) | GDSL-like Lipase/Acylhydrolase | Carbohydrate Esterase Family 3 |
| 43373 | gw1.10.153.1 | scaffold_10:997206-998495 (+) | GDSL-like Lipase/Acylhydrolase | hydrolase activity on ester bonds |
| 72612 | e_gw1.1.2278.1 | scaffold_1:1349400-1350411 (-) | GDSL-like Lipase/Acylhydrolase | Carbohydrate Esterase Family 3 |
| 92434 | e_gw1.11.353.1 | scaffold_11:1065788-1067065 (-) | GDSL-like Lipase/Acylhydrolase | Carbohydrate Esterase Family 3 |
| 509409 | fgenesh1_kg.8_#_31_#_Locus8614v1rpkm2.33 | scaffold_8:113072-113949 (-) | GDSL-like Lipase/Acylhydrolase | Isoamyl acetate-hydrolyzing esterase |
| 510248 | fgenesh1_kg.10_#_8_#_Locus10596v1rpkm1.04 | scaffold_10:53541-54634 (+) | GDSL-like Lipase/Acylhydrolase | Carbohydrate Esterase Family 3 |
| 516582 | estExt_fgenesh1_pm.C_2_t30018 | scaffold_2:2892227-2893894 (+) | GDSL-like Lipase/Acylhydrolase | Isoamyl acetate-hydrolyzing esterase |
| 85395 | e_gw1.6.337.1 | scaffold_6:679883-681325 (-) | Predicted alpha/beta hydrolase | DUF676_lipase-like |
| 100838 | e_gw1.29.72.1 | scaffold_29:106446-107981 (+) | Lipase_GDXG_HIS_AS, AB_hydrolase | Arylacetamide deacetylase |
| 118553 | estExt_Genewise1Plus.C_8_t10171 | scaffold_8:191543-195600 (-) | GDSL-like Lipase/Acylhydrolase | Carbohydrate Esterase Family 3 |
| 157348 | estExt_Genewise1.C_490011 | scaffold_49:20234-23599 (+) | GDSL-like Lipase/Acylhydrolase | Carbohydrate Esterase Family 3 |
| 514427 | fgenesh1_kg.83_#_1_#_Locus825v1rpkm186.18 | scaffold_83:2335-3893 (+) | Lipase_secreted | Secretory lipase; iron ion transport |
| 77612 | e_gw1.3.1367.1 | scaffold_3:1583837-1587250 (-) | Phospholipase C | Phosphoinositide-specific phospholipase C |
| 222426 | CE52577_10851 | scaffold_11:194867-198458 (+) | Phospholipase C | Phosphoinositide-specific phospholipase C |
| 482782 | fgenesh1_pg.7_#_388 | scaffold_7:1132150-1133895 (+) | Phospholipase C | Phosphoinositide-specific phospholipase C |
| 490471 | fgenesh1_pm.2_#_392 | scaffold_2:1112245-1114326 (-) | Phospholipase C | Phosphoinositide-specific phospholipase C |
| 536904 | MIX577_51_94 | scaffold_1:739432-741727 (-) | Lipase_GDXG_HIS_AS,  CarbesteraseB | Carboxylesterase family |
| 91073 | e_gw1.10.999.1 | scaffold_10:814005-815072 (-) | Phospholipase C | Glycosylphosphatidylinositol-specific phospholipase C |
| 481511 | fgenesh1_pg.5_#_682 | scaffold_5:2279112-2280468 (+) | Phospholipase C | PLipase_C_PInositol |
| 504276 | fgenesh1_kg.2_#_820_#_Locus1919v1rpkm78.8 | scaffold_2:2472619-2474504 (+) | Phospholipase C | PLipase_C_PInositol |
| 509413 | fgenesh1_kg.8_#_35_#_Locus4260v1rpkm24.39 | scaffold_8:129174-131043 (+) | Phospholipase C | Glycosylphosphatidylinositol-specific phospholipase C |
| 72504 | e_gw1.1.3340.1 | scaffold_1:3946901-3949351 (-) | Acyl_Trfase/lysoPLipase | Lysophospholipase |
| 350643 | CE180794_97 | scaffold_3:1591594-1592361 (-) | Phospholipase A2 | Phospholipid metabolic process |
| 443708 | CE273859_156 | scaffold_7:624825-625362 (-) | Phospholipase A2 | Phospholipid metabolic process |
| 449569 | CE279720_76 | scaffold_7:1520373-1520871 (-) | Phospholipase A2 | Phospholipid metabolic process |
| 549088 | MIX12761_545_96 | scaffold_3:3077745-3079308 (+) | Phosphatidylinositol phospholipase | Lipid metabolic process, oxidoreductase activity |
| 77540 | e_gw1.3.2811.1 | scaffold_3:1671663-1673855 (+) | Sphingomyelin phosphodiesterase | Acid sphingomyelinase and PHM5 phosphate metabolism |
| 93268 | e_gw1.12.65.1 | scaffold_12:840535-842776 (-) | Acyl_Trfase/lysoPLipase | Lysophospholipase |
| 514118 | fgenesh1_kg.31_#_48_#_Locus5221v1rpkm15.36 | scaffold_31:139768-142584 (+) | Acyl_Trfase/lysoPLipase | Lysophospholipase |


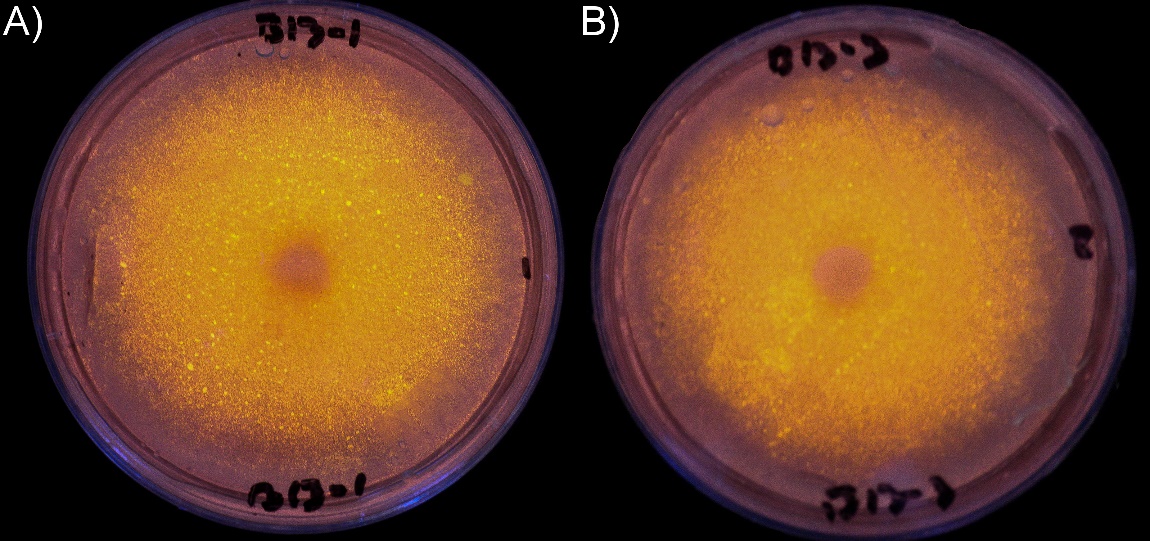


**Figure S1:** Fungal strains B13-1 (A) and B13-3 (B) selected in screening for extracellular lipolytic activity using olive oil as carbon source in Rhodamine B test.


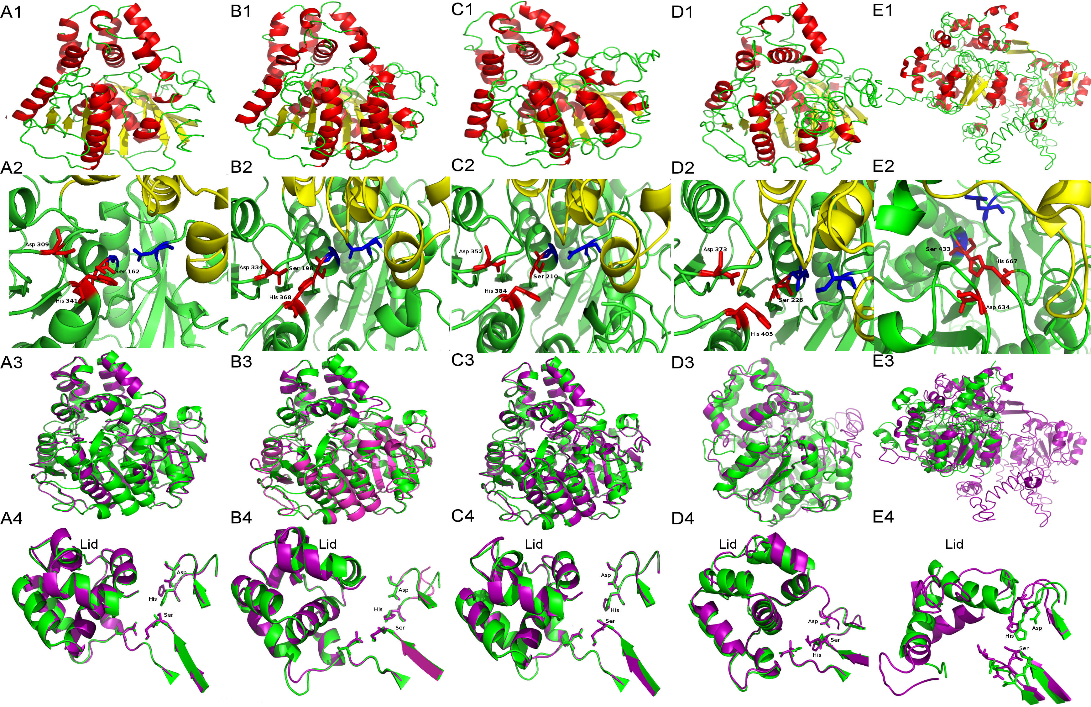

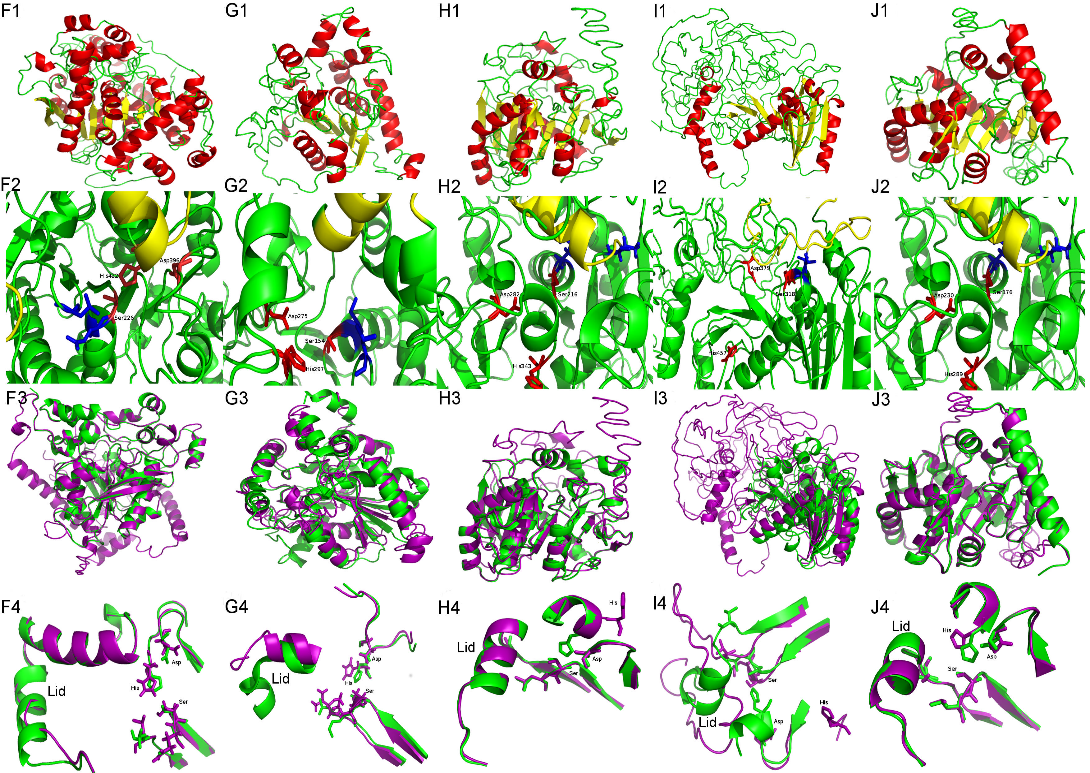


**Figure S2:** Three-dimensional model proposed for (A) 551811; (B) 87496; (C) 526309; (D) 514427; (E) 510832; (F) 92423; (G) 135964; (H) 77338; (I) 514252; (J) 78181. In all protein models: (1) Red, α-helixes; yellow, β-strands. (2) Close up showing the lid (yellow), the catalytic triad (in red) and the oxyanion (in blue). (3) Superposition with best template protein model (see Table 4). (4) Superposition of catalytic triad in both proteins. All models were generated by I-TASSER and visualizations were performed in PyMOL.
